# Comparing random forest and elastic net models to predict substance use disorder transitions in participants with cannabis and stimulant use: Evidence from the All of Us cohort

**DOI:** 10.1016/j.drugalcdep.2025.113012

**Published:** 2025-12-18

**Authors:** Gabriel Zamora, Tommy Gunawan, Qingyu Zhao, Alejandro D. Meruelo

**Affiliations:** a University of California, San Diego, 9500 Gilman Dr La Jolla, CA 92093, USA; b Department of Medical and Clinical Psychology, Uniformed Services University of the Health Sciences, Bethesda, MD 20814, USA; c Henry M. Jackson Foundation for the Advancement of Military Medicine, Bethesda, MD 20817, USA; d Department of Radiology, Weill Cornell Medicine, New York, NY 10065, USA

**Keywords:** Random forest, Elastic net, Machine learning, Cannabis, Stimulants, Social determinants of health, Wearable data

## Abstract

**Background:**

Predicting progression from substance use to substance use disorder (SUD) is challenging, particularly for participants with cannabis and stimulant use who follow distinct risk trajectories. Machine learning enables integration of demographic, behavioral, wearable-derived, and social determinants of health (SDoH) data, yet few studies have compared linear and non-linear approaches in large, diverse populations.

**Methods:**

Data came from the All of Us Research Program, a nationwide cohort integrating electronic health records, surveys, wearable metrics, and SDoH. Individuals with baseline cannabis or stimulant use were followed for incident SUD diagnoses. Predictors included demographics, wearable-derived activity and sleep, and SDoH domains (income, food insecurity, housing instability, transportation barriers). Elastic net (EN) logistic regression and random forest (RF) models were trained separately within cannabis and stimulant cohorts. Discrimination was evaluated on independent test sets using the area under the receiver operating characteristic curve (AUC) and compared with the DeLong test.

**Results:**

For participants with cannabis use, EN and RF showed similar performance (AUC = 0.740 vs. 0.741; DeLong p = 0.764). For participants with stimulant use, RF achieved AUC = 0.732 vs. EN = 0.698; DeLong p = 0.219. Demographic variables were the strongest predictors across models. SDoH indicators—particularly income—contributed substantially to prediction, while wearable-derived metrics provided incremental explanatory value primarily in EN models, with limited independent contribution in RF.

**Conclusions:**

EN and RF models achieved moderate prediction of SUD transitions. Incorporating SDoH and wearable-derived data enhanced interpretability and risk stratification, particularly in linear models, underscoring substance-specific pathways and the utility of multimodal frameworks for developing precision prevention strategies.

## 1. Introduction

Substance use disorders (SUDs) remain a significant public health challenge, contributing to substantial morbidity, mortality, and socioeconomic costs (Connery et al., 2020; Jain et al., 2021; Substance use disorders, 2022). Among the most commonly used substances, cannabis (Hasin et al., 2019; Volkow et al., 2016b; Wilkinson et al., 2016) and stimulants (Connery et al., 2020; Degenhardt and Hall, 2012), including cocaine and amphetamines, present distinct but equally urgent challenges for prevention and treatment. Cannabis use has risen markedly in recent years, driven in part by legalization trends (Wilkinson et al., 2016), while stimulant use, though less prevalent overall, has been accompanied by a sustained rise in stimulant-involved overdose deaths over more than a decade. National surveillance reports indicate that cocaine-involved overdose deaths increased from roughly 5000 deaths in 2010 to over 24,000 deaths in 2021, and psychostimulant-involved deaths (including amphetamine and methamphetamine) rose from under 2,000 to more than 32000 over the same period, reflecting multi-year escalation rather than a single-year aberration (Hedegaard, 2020; Spencer et al., 2023). Stimulant use remains strongly associated with elevated risks of dependence, overdose, and psychiatric comorbidities (Connery et al., 2020; Hedegaard, 2020). Much of the existing literature has focused on treatment outcomes and epidemiologic trends (Jain et al., 2021), with relatively less attention paid to identifying predictors of transition from substance use to SUD in large, population-based samples.

Growing evidence suggests that demographic characteristics, behavioral factors, and physiological patterns (Kendler et al., 2019; Sussman and Sussman, 2011)—such as physical activity (Terry-McElrath and O’Malley, 2011) and sleep metrics (Hasler et al., 2016; Wong et al., 2015)—play important roles in the onset and progression of SUDs. Importantly, understanding the drivers of progression to SUD can help identify individuals most at risk and inform early, targeted interventions. In addition, social determinants of health (SDoH), including socioeconomic status, food insecurity, housing instability, and limited access to healthcare, have been increasingly recognized as key drivers of substance-related disparities (Alegría et al., 2015; Galea and Vlahov, 2002; Marmot and Bell, 2012). Despite this, few predictive modeling studies have explicitly integrated SDoH alongside behavioral and clinical measures, leaving important gaps in understanding the pathways through which structural disadvantage shapes SUD risk. Advances in wearable technologies and the integration of electronic health records (EHR) with survey and environmental data now make it possible to track both biological and contextual predictors with high resolution and at scale. However, prior modeling efforts have often been constrained by limited demographic diversity, small sample sizes, or reliance on self-reported outcomes, restricting generalizability.

The All of Us Research Program (Denny et al., 2019) provides a uniquely powerful resource to overcome these limitations, offering a large, diverse, and deeply phenotyped cohort with linked survey, EHR, SDoH, and wearable data. Despite the richness of this dataset, no study to date has systematically evaluated machine learning–based models to predict SUD transition specifically for participants with cannabis and stimulant use, nor compared predictive performance and feature importance across these substances in a unified analytic framework. Machine learning approaches are particularly well-suited for this task, as they can accommodate high-dimensional, multimodal data and detect complex, non-linear interactions among biological, behavioral, and contextual factors that may be missed by traditional analytic methods.

The present study applies complementary modeling strat- egies—elastic net regression (Zou and Hastie, 2005), which allows for interpretable feature selection, and random forest (Aria et al., 2021; Breiman, 2001; Schonlau and Zou, 2020), which captures non-linear relationships—to predict transition to SUD in baseline cannabis and stimulant cohorts. The goal was to evaluate the ability of these models to discriminate future SUD cases, identify the most influential demographic, behavioral, and SDoH predictors, and assess whether their relative importance differed between substance types. Comparing elastic net and random forest is clinically meaningful because elastic net facilitates identification of a concise set of interpretable predictors that can directly inform targeted prevention strategies, while random forest allows for the detection of complex, non-linear interactions that may highlight high-risk subgroups invisible to simpler models, together offering complementary insights for policy and intervention design.

We hypothesized that models would achieve moderate discrimination and that demographic variables such as age, race (Substance Abuse and Mental Health Services Administration, 2024), and sex at birth (Volkow and Blanco, 2023) would emerge as strong predictors across cohorts, given their well-established associations with both substance initiation and progression to SUD in prior epidemiological studies. We further anticipated that integrating SDoH domains—particularly income, food insecurity, and transportation barriers—would explain additional variance in SUD transitions beyond demographics and behavioral measures by capturing environmental and structural risk factors not reflected in individual-level characteristics. Finally, we expected that physical activity and sleep patterns derived from wearable devices would provide complementary predictive value and that feature importance would vary between cannabis and stimulant cohorts, reflecting substance-specific behavioral and social risk profiles.

## 2. Methods

### 2.1. Data source and study population

Data for this study were obtained from the All of Us Research Program version 8 (Denny et al., 2019), a large, longitudinal, and demo- graphically diverse cohort that integrates electronic health records (EHR), survey data, biospecimens, and digital health information. Participants provided informed consent and authorized linkage of their EHR and wearable device data. The analytic sample included individuals with documented cannabis or stimulant use at baseline, identified through relevant concepts in the Observational Medical Outcomes Partnership (OMOP) common data model condition_occurrence table, mapped from SNOMED (Systematized Nomenclature of Medicine) codes. OMOP provides a standardized data structure for harmonizing EHR information across healthcare systems, while SNOMED is a comprehensive clinical terminology that encodes medical concepts such as “Cannabis use” or “Stimulant intoxication.” This framework enables consistent identification of diagnostic and clinical events across sites. Cannabis and stimulant cohorts were defined using EHR curated concept ID lists capturing both use and use disorder diagnoses, as well as related clinical presentations such as intoxication, withdrawal, and overdose. Participants with pre-existing cannabis or stimulant use disorder diagnoses prior to baseline were excluded to ensure that all cases represented incident onsets of substance-specific SUD during follow-up. Participants were uniquely assigned to a primary substance cohort via a hierarchical rule based on earliest documented use. Standardized effect sizes were calculated for demographic characteristics (Table 1) (Cohen, 1988). In addition, to quantify potential wearable-participation bias, we compared baseline characteristics for participants with versus without any Fitbit data and reported standardized mean differences (SMDs) overall and by cohort.

To address possible exposure misclassification in the stimulant cohort related to therapeutic use for conditions such as attention-deficit/ hyperactivity disorder and narcolepsy, we analyzed three stimulant specifications: a Primary set including all stimulant-coded exposure at baseline, a Flagged set identical to Primary but with an indicator for probable therapeutic-use contexts, and a Strict set that excluded probable therapeutic-use patterns in the absence of misuse codes. This design isolates non-medical exposure and supports sensitivity analyses without altering the cannabis definition.

#### 2.1.1. Outcome definition

The primary outcome was incident SUD related to the baseline substance category (cannabis or stimulants) after study entry. Cases were identified using OMOP condition concept IDs for dependence, abuse, and related syndromes. Outcomes were assessed prospectively from baseline until first SUD diagnosis or censoring. Among non-cases, median follow-up was ~730 days; among cases, median time-to-event was 111 days for cannabis and 33 days for stimulants.

For survival sensitivity analyses, we constructed time-to-event records from index (baseline) to the first substance-specific SUD diagnosis or to the last available follow-up for non-cases (administrative censoring at ~730 days). For survival models, we defined time in days from index to event or censoring and an event indicator (1 = incident SUD, 0 = censored).

#### 2.1.2. Predictor variables

Predictors included demographic variables (age at baseline, sex at birth, gender identity, race, ethnicity), social determinants of health (SDoH) from All of Us surveys (household income, food insecurity, transportation barriers, housing instability, internet access, education, non-English language at home), and wearable-derived features summarizing physical activity, heart rate, and sleep. Fitbit variables included mean and variability of daily steps, sedentary and active minutes, time in heart rate zones, proportion of days with zero steps, total sleep minutes, and sleep continuity indices.

Survey data supplemented EHR-derived race and ethnicity. Wearable data were summarized into person-level baseline features by averaging and computing variability across valid days in a predefined baseline window, so each participant contributed a single value per wearable predictor. Continuous variables were standardized; categorical variables were encoded as factors. Missing categorical values were retained as explicit “missing” levels; continuous predictors were analyzed on complete cases when constructing design matrices.

For elastic net models, categorical predictors were one-hot encoded; random forest used them as single multi-level factors. The same predictor set was used for time-to-event sensitivity analyses with Cox elastic net and Random Survival Forests after aligning train–test design matrices. Fitbit participation (“has Fitbit at baseline”) was defined as any valid Fitbit record in the baseline window and was used only for SMD summaries and inverse-probability weighting (IPW).

Before preprocessing, continuous Fitbit predictors had high missingness (94–96 %) because only a subset of participants had linked devices, whereas categorical predictors had low to moderate missingness (e.g., sex at birth Unknown = 0.74 %). After factor encoding and complete-case restriction for continuous variables, the analytic design matrices contained no missing values. Detailed descriptive statistics and missingness patterns are provided in Supplementary Table S1.

### 2.2. Data processing

For each cohort, we restricted analyses to participants with complete outcome and predictor information in the final design matrices. Predictors with near-zero variance were removed (minority class *<*1 % for categorical variables or variance *<*1e-5 for continuous variables) to avoid unstable estimates and reduce noise. A small number of sparsely endorsed SDoH variables (e.g., housing instability, intimate partner violence) were excluded for this reason; all retained predictors demonstrated sufficient variability (Table 2).

The analytic datasets were split into training (70 %) and testing (30 %) sets using stratified random sampling based on the binary outcome to preserve case–control proportions. The same split was reused for time-to-event sensitivity analyses. All model-based interpretability summaries (partial dependence, interactions, permutation importance) were computed on training data and exported only as aggregate summaries. All analyses were performed in R 4.4.3 (R Core Team, 2021) within the All of Us Workbench.

### 2.3. Modeling approach

We fit two supervised learning models in each cohort: elastic net (EN) logistic regression (Zou and Hastie, 2005) and random forest (RF) classification (Breiman, 2001). Elastic net was chosen for its ability to perform variable selection and handle multicollinearity, and random forest for its capacity to capture non-linear relationships and interactions. Elastic net models were implemented with glmnet (Friedman et al., 2010) and tuned via nested 10-fold cross-validation over a grid of α values; within each inner loop, λ was selected by minimum cross-validated deviance. Random forest models were implemented with ranger (Wright and Ziegler, 2017) using probabilistic outputs; mtry and minimum node size were tuned by 5-fold cross-validation, with 400 trees per model. Class imbalance was addressed using inverse-frequency class weights, and cannabis and stimulant cohorts were modeled separately.

For stimulants, we estimated three prespecified model specifications to address potential therapeutic prescribing. The Primary specification included all participants with stimulant-coded exposure at baseline. The Flagged specification retained the same cohort but added an indicator for probable therapeutic exposure. The Strict specification excluded participants whose stimulant patterns were consistent with therapeutic use without misuse codes, thereby enriching for probable non-medical use at the cost of a smaller sample.

As sensitivity analyses for censoring, we fit penalized Cox models with an elastic-net penalty and Random Survival Forests (RSF) (Ishwaran and Kogalur, 2025) using the same predictors and train–test split. Cox models used cross-validated λ selection, whereas RSF used several hundred trees with standard settings to obtain cumulative hazard–based risk scores. For interpretability, we computed mean-only partial dependence (PD) curves (Greenwell, 2017) for selected predictors and Friedman’s H interaction scores derived from two-way PD surfaces. We estimated permutation-based random forest feature importance by repeatedly permuting each predictor in the held-out test set and quantifying the resulting mean decrease in AUC, with results summarized as cohort-level tables.

### 2.4. Model evaluation

Primary evaluation was based on area under the receiver operating characteristic curve (AUC) on held-out test sets, with EN–RF differences compared using DeLong’s method (Zhu et al., 2024). For stimulants, we additionally computed area under the precision–recall curve (PR-AUC) with bootstrap confidence intervals, given the low outcome prevalence. Threshold-based metrics were summarized at the Youden J index and at a fixed top-k proportion to reflect capacity-limited screening scenarios.

For survival sensitivity analyses, we reported Harrell’s C-index (Uno et al., 2011) to assess rank discrimination and time-dependent AUC at 365 and 600 days (Blanche et al., 2013). Uno’s integrated AUC over 0–600 days was computed to summarize discrimination across time (Potapov et al., 2025; Uno et al., 2007).

To assess robustness to differential wearable participation, we estimated IPW (Chesnaye et al., 2021) for “has Fitbit” using logistic regression on demographics and SDoH (age, sex at birth, gender, race, ethnicity, income, food insecurity, transportation barriers, non-English language, and internet access). Weighted AUC and PR-AUC were then recomputed on the test sets with bootstrap confidence intervals. IPW was used only for these robustness checks.

### 2.5. Multicollinearity assessment (Supplementary Figures S1 & S2)

We assessed multicollinearity using variance inflation factors (VIFs), pairwise Pearson correlations, and the overall condition number of the design matrix. Predictors with |r| ≥ 0.90 were flagged as highly correlated. To evaluate the impact of collinearity, we re-fit models after (i) removing one variable from each highly correlated pair and (ii) replacing correlated predictors with principal components explaining ≥ 95 % of variance. Survival sensitivity analyses used the original feature set. Multicollinearity diagnostics did not alter the interpretability pipeline.

### 2.6. Fairness analysis

We examined racial and ethnic fairness using predicted positive rate (PPR), false positive rate (FPR), and false negative rate (FNR) at the Youden J and top-k thresholds after isotonic calibration, following an equalized-odds perspective (Hardt et al., 2016). Subgroup metrics were summarized with 95 % bootstrap confidence intervals and compared with White participants as a reference. Subgroup calibration was assessed with expected calibration error and reliability curves. Fairness analyses were conducted in the binary classification setting without IPW, so results reflect the observed test distribution.

## 3. Results

### 3.1. Study cohorts

We identified 146,800 participants with relevant condition data. At cohort entry, 142,730 were classified into the cannabis cohort and 4070 into the stimulants cohort. Within two years, 2298 cannabis participants (1.61 %) and 94 stimulant participants (2.31 %) developed a substancespecific SUD. Across both cohorts, 2298 participants (1.57 %) developed cannabis-only SUD, 94 (0.064 %) developed stimulant-only SUD, none developed both, and 144,408 (98.4 %) developed neither. Full demographic summaries and SMDs (including with-Fitbit vs no-Fitbit comparisons) are provided in Supplementary Table S2.

To address therapeutic stimulant exposure, we analyzed the prespecified Strict and Flagged stimulant specifications. The Strict definition excluded likely participants with therapeutic use without misuse codes, modestly reducing the stimulant analytic set (overall n from 4048 to 3907; held-out test set n = 782 with 19 events) and leaving sex distribution essentially unchanged (female 70.7 % → 70.9 %).

### 3.2. Model performance (Figs. 1 and 2)

In the cannabis cohort, EN and RF achieved similar discrimination: AUC_EN = 0.740 (95% CI: 0.718–0.761) and AUC_RF = 0.741 (95 % CI: 0.719–0.763), with no significant difference (DeLong p = 0.764). Both models showed moderate discrimination and comparable sensitivity–specificity profiles.

In the stimulant cohort, AUC_EN = 0.698 (95 % CI: 0.586–0.810) and AUC_RF = 0.732 (95 % CI: 0.624–0.840); the difference was not statistically significant (p = 0.219) but suggested somewhat better nonlinear performance in the smaller stimulant sample. Both models showed stable precision–recall behavior, with RF offering slightly higher sensitivity at comparable specificity (Supplementary Table S3; Supplementary Figure S3).

Survival sensitivity analyses produced broadly consistent patterns. In cannabis, Cox elastic net and Random Survival Forest achieved Harrell’s C of 0.725 and 0.694, respectively; time-dependent AUC at 1 year was modest (0.276 and 0.305), with Uno’s iAUC0–600d of 0.274 and 0.304. In stimulants, C-indices were 0.776 (Cox-EN) and 0.772 (RSF), with 1- year tdAUC ≈ 0.23 and iAUC0–600d ≈ 0.22–0.23. These results are consistent with low event rates and administrative censoring and support the robustness of the primary binary analyses.

In the Strict stimulant cohort, discrimination improved for both models (AUC_EN =0.795, 95 % CI: 0.691–0.886; AUC_RF =0.799, 95 % CI: 0.692–0.895; DeLong p = 0.836). PR-AUCs also increased (0.091 for EN and 0.173 for RF), indicating better early retrieval despite low prevalence. In the Flagged cohort, AUCs were 0.692 (EN) and 0.735 (RF) (p = 0.168). Precision–recall curves for all specifications are shown in Supplementary Figure S4.

IPW-reweighted test-set AUC and PR-AUC closely matched the unweighted results for all model–cohort combinations, with overlapping bootstrap confidence intervals (Supplementary Table S4), suggesting that differential wearable participation did not materially bias discrimination estimates.

### 3.3. Feature importance and model comparison

**Fig. 3.**
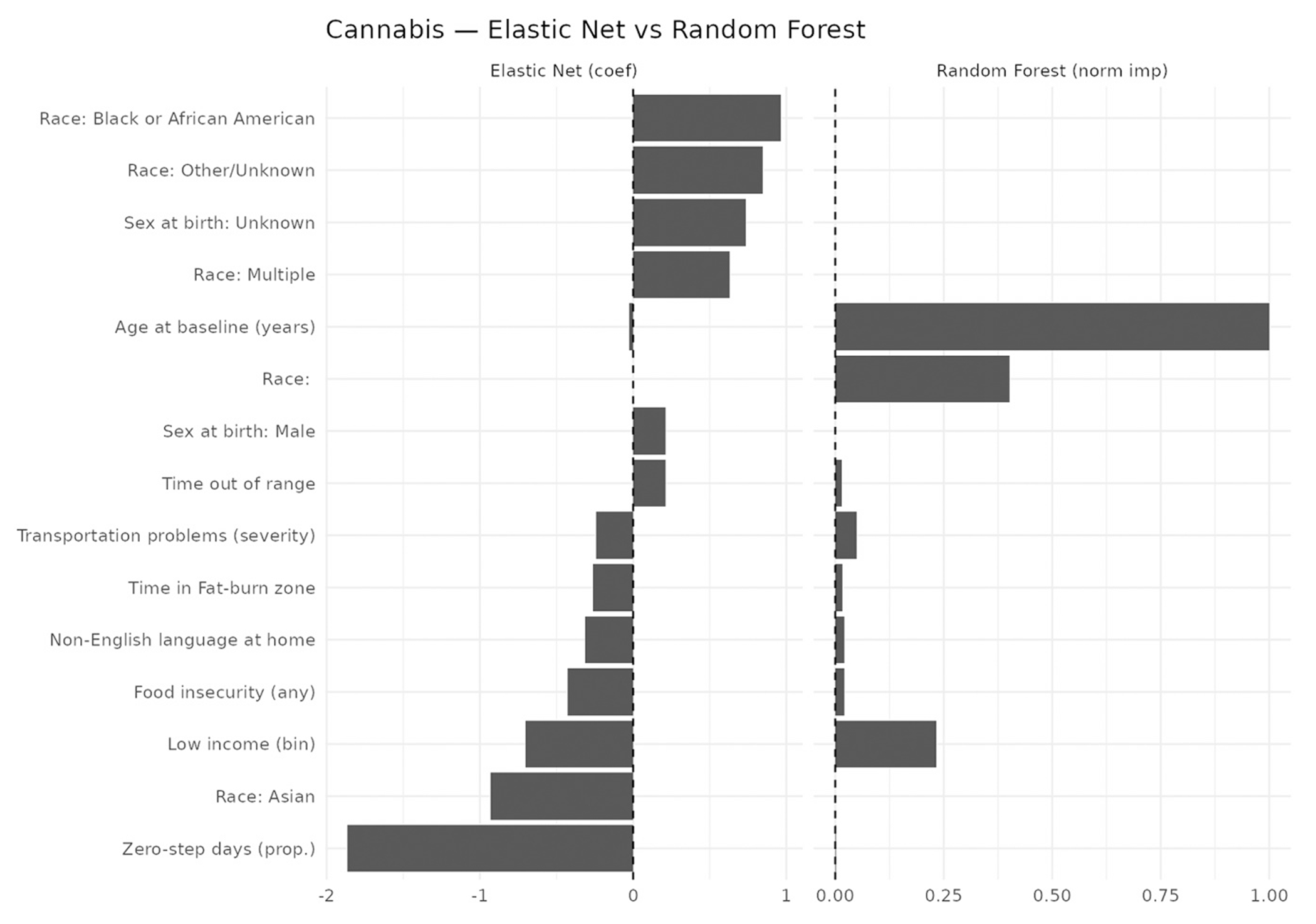
Comparison of Elastic Net Coefficients and Random Forest Importances for Cannabis.Comparison of elastic net coefficients (left; showing effect size and direction) and random forest normalized permutation importances (right) for cannabis-related transition prediction. Elastic net highlights directional linear associations across a broad set of predictors, including demographic characteristics, physical activity metrics (e.g., zero-step days, time spent in peak and cardio heart-rate zones), and selected social determinants of health. In contrast, random forest permutation importance indicates that predictive performance is dominated by core demographic variables—particularly age, sex at birth, and race—with household income contributing modestly, while most activity-derived and secondary SDoH variables show minimal incremental predictive value. Together, these results illustrate how elastic net captures distributed linear associations, whereas random forest emphasizes predictors that uniquely improve non-linear out-of-sample discrimination.

For cannabis (Fig. 3), both EN (Supplementary Table S5) and RF emphasized demographic and socioeconomic predictors (age, race, sex at birth, income). EN provided directional effects, including higher predicted transition risk among Black or African American participants and lower risk associated with Asian race and greater physical activity (e.g., fewer zero-step days, more time in higher heart rate zones). RF identified a more concentrated set of dominant predictors, with permutation-tested importance indicating that age, race, sex at birth, gender identity, and low income accounted for most of the model’s predictive signal. In contrast, wearable-derived activity measures and secondary SDoH variables contributed little incremental predictive value in RF and were not statistically significant in permutation testing (Supplementary Cannabis Table S6).

Mean-only PD curves indicated decreasing risk with older age, with a candidate threshold near 40 years, and higher predicted risk associated with low income. Race, sex, and gender showed level differences without strong non-monotonicity (Supplementary PDP Table S7).

**Fig. 4.**
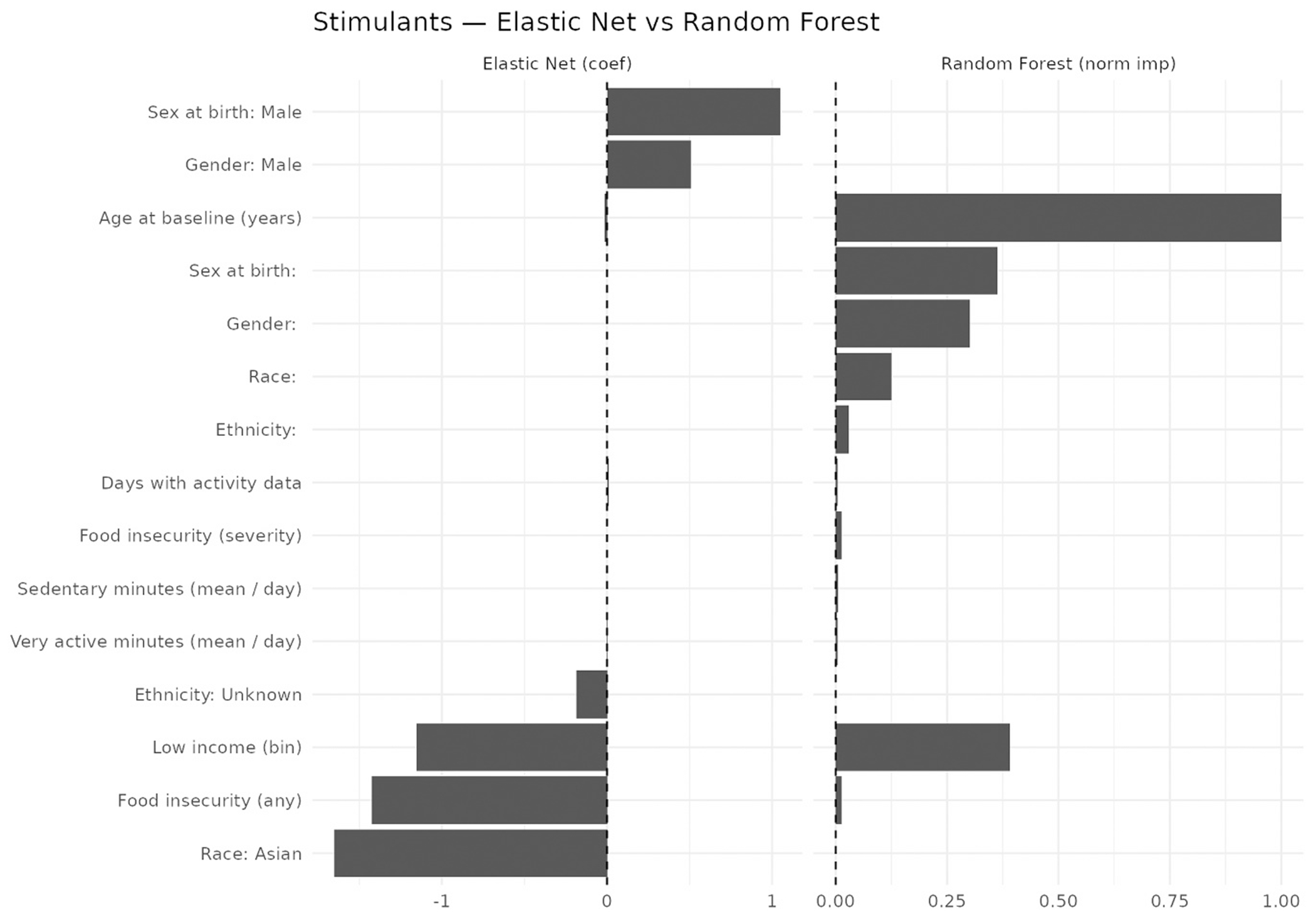
Comparison of Elastic Net Coefficients and Random Forest Importances for Stimulants.Comparison of elastic net coefficients (left; showing effect size and direction) and random forest normalized permutation importances (right) for stimulant-related transition prediction. Elastic net identifies directional linear associations across multiple domains, including demographic characteristics and selected social determinants of health, such as sex at birth, race, and household income. In contrast, random forest permutation importance indicates that predictive performance is driven primarily by core demographic variables—particularly age, sex at birth, gender identity, and household income—while most activity-derived measures and secondary SDoH variables contribute little additional predictive value. These results highlight complementary model behavior: elastic net captures distributed linear associations across correlated predictors, whereas random forest emphasizes variables that uniquely improve non-linear predictive discrimination.

For stimulants (Fig. 4), both models identified male sex, gender identity, age, and low income as primary drivers of risk. EN suggested lower predicted transition risk for Asian race and some low-income or food-insecure categories (Supplementary Table S5), whereas RF emphasized a narrow set of predictors—primarily sex at birth, gender identity, and low income—as the main contributors to predictive performance. Although elastic net identified directional associations involving additional activity and sleep variables, permutation-based RF importance indicated that these features provided minimal incremental predictive value beyond core demographic and income measures (Supplementary Stimulant Table S8). Age did not reach statistical significance in RF permutation testing. PD curves suggested increasing risk with age and higher risk in low-income groups, with candidate age thresholds in the mid-40s (Supplementary PDP Table S9).

In the Strict stimulant cohort, EN selected approximately 10 nonzero predictors (vs. ~8 in the primary and Flagged specifications), but performance gains appeared driven primarily by the cleaner exposure definition rather than increased model complexity, and the selected predictors were predominantly demographic and SDoH features (Supplementary Table S5, Supplementary Figure S5).

Taken together, these results suggest that while elastic net captures broader linear associations across correlated social and behavioral features, random forest prediction in this setting is driven primarily by a smaller subset of core demographic and socioeconomic variables, highlighting that not all associated predictors contribute substantively to non-linear predictive performance.

### 3.4. Multicollinearity diagnostics

The overall condition number indicated moderate multicollinearity (κ ≈ 15–17). Elastic net performance and coefficient patterns were highly consistent under correlation-pruned and PCA-transformed predictor sets, with minimal changes in AUC (Supplementary Figure S6). RF performance, particularly in the stimulant cohort, degraded when correlated predictors were replaced by principal components, suggesting that RF benefited from directly modeling the original correlated features. Because stimulant events were rare, EPV for EN in that cohort was low (~2), so penalization and bootstrap intervals were emphasized, and causal interpretation was avoided.

### 3.5. Fairness analysis (Supplementary Figures S7–S8; Supplementary Table S10)

Fairness analyses, conducted by race and ethnicity using predicted positive rate (PPR), false positive rate (FPR), and false negative rate (FNR) at the Youden J and Top-k operating points after post-hoc isotonic calibration, revealed notable racial and ethnic disparities for cannabis models. Black, Multiple, and Other/Unknown participants showed markedly higher predicted positive rates than White participants (PPR Δ ≈ +50–70 %) and higher FPR at both thresholds, whereas Asian participants were consistently under-predicted, in part reflecting small subgroup sizes. Isotonic calibration yielded acceptable overall calibration and brought subgroup reliability curves closer to the diagonal, but did not remove performance gaps; curves remained noisier in smaller categories.

For stimulants, racial disparities were smaller but still present. At the validation-selected thresholds, PPR and FPR were modestly higher in Black and Other/Unknown groups and lower in White and Asian groups, with similar patterns for Hispanic/Latino versus Unknown ethnicity. These results held under the Strict stimulant definition, which increased sensitivity but also raised FPR. Calibration differences by model were notable: after isotonic calibration, elastic net tended to remain overconfident across subgroups (race ECE ≈ 0.34–0.45; ethnicity ECE ≈ 0.36–0.38), whereas random forest was better calibrated (race ECE ≈ 0.002–0.03; ethnicity ECE ≈ 0.002–0.03), with reliability curves closer to the diagonal. Given the low stimulant SUD prevalence, very small subgroup event counts in held-out folds (≈2–3 %; ~19 events per split), and cohort base-rate differences, these subgroup estimates are best interpreted as diagnostic rather than definitive.

## 4. Discussion

This study leveraged the All of Us Research Program to predict SUD transitions among adults with baseline cannabis or stimulant use, integrating demographic, SDoH, and wearable-derived behavioral features. Both EN and RF models achieved moderate discrimination, consistent with prior work using demographic and clinical predictors alone (Hasin et al., 2019; Saloner and Leˆ Cook, 2013), and demonstrated that incorporating SDoH and wearable metrics can modestly improve prediction primarily in elastic net models while highlighting substance-specific risk pathways. IPW analyses indicated that differential Fitbit participation did not materially bias discrimination, and time-to-event sensitivity analyses corroborated the primary binary results despite administrative censoring.

### 4.1. Demographic and socioeconomic predictors

Demographic variables—particularly age, sex at birth, and race—were among the strongest predictors of SUD transitions, aligning with prior epidemiologic research (Alvanzo et al., 2014; Chen et al., 2009; Compton et al., 2007). Our findings extend this literature by demonstrating that their relative contributions differ between cannabis and stimulant cohorts.

Unlike many population surveys in which males report higher cannabis and stimulant use, baseline cannabis and stimulant use in this EHR-derived All of Us sample was more common among females (Table 1). Because these cohorts are defined by health system encounters and voluntary research participation rather than population sampling, this pattern likely reflects differences in healthcare utilization, screening, and diagnostic capture—where women are often over- represented—rather than a true reversal of population-level sex differences in substance use (Bertakis et al., 2000).

For cannabis, both EN and RF highlighted elevated predicted transition risk among Black or African American participants (Pacek et al., 2020; Wu et al., 2016) and lower predicted risk among Asian participants and those reporting non-English language use, consistent with reports of lower cannabis prevalence and delayed onset in some Asian and first-generation immigrant groups (Wu et al., 2014). For stimulants, male sex and gender identity exerted strong influences on transition risk, consistent with higher stimulant escalation rates among men (Compton et al., 2007), but EN suggested somewhat lower predicted risk in some low-income and food-insecure categories (Cerdá et al., 2018; Saloner and Lˆe Cook, 2013). The divergence between EN and RF for these socioeconomic predictors, and the prominence of income and food insecurity in RF permutation importance, suggest that non-linear thresholds or interactions between structural disadvantage and other factors may shape stimulant-related risk in complex ways (Galea and Vlahov, 2002; Marmot and Bell, 2012). At the same time, RF permutation testing indicated that income-related variables dominated model importance, with other SDoH contributing little independent predictive signal. Time-to-event models reinforced the dominant role of demographic and socioeconomic structure rather than short-term wearable signals.

### 4.2. Behavioral features and substance-specific pathways

Wearable-derived activity and sleep measures provided a complementary behavioral dimension. Prior research has linked disrupted circadian rhythms, reduced physical activity, and poor sleep to higher substance use vulnerability (Hasler et al., 2015), and our findings are broadly consistent with this pattern. While overall Fitbit coverage was limited and participants with wearables differed from those without, IPW-adjusted discrimination estimates closely matched unweighted results, supporting robustness to wearable-participation bias in ranking performance.

For cannabis transitions, EN indicated that greater time in fat-burn and cardio zones and fewer zero-step days were associated with lower predicted risk, possibly reflecting underlying social and occupational stability rather than causal protection. RF identified similar behavioral predictors in rank order but ranked them below key demographic and SDoH variables, and permutation testing suggested that some wearable fractions contributed less than demographic features and were not statistically significant contributors. Survival results showed good overall rank discrimination but modest early-horizon AUCs, implying that wearable-linked behaviors may contribute more to long-term stratification than to very early event prediction.

For stimulants, behavioral features played a smaller role in EN models, with most predictive signal carried by demographic and socioeconomic variables. RF, however, ranked activity and sleep features far below demographic and income variables, and permutation testing indicated no statistically significant independent contribution of these behavioral measures, underscoring the primacy of structural and demographic factors in stimulant-related risk.

Together, these results suggest that behavioral disruptions may represent substance-specific risk signals, consistent with emerging work on differential pathways to cannabis- versus stimulant-related SUDs (Koob and Volkow, 2016; Parlatini et al., 2024; Volkow et al., 2016a; Zehra et al., 2018).

### 4.3. Model comparisons and methodological insights

Comparing EN and RF illustrates how linear and non-linear modeling approaches provide complementary insights. Notably, several behavioral and SDoH variables retained directional importance in EN but showed minimal or null contribution in RF permutation testing. For cannabis, EN and RF performed nearly identically, suggesting that much of the signal can be captured with relatively simple linear effects and limited interactions. For stimulants, RF showed a modest, nonsignificant AUC advantage, consistent with an outcome influenced by heterogeneous and potentially interaction-driven mechanisms (Breiman, 2001; Couronné et al., 2018).

Survival sensitivity analyses yielded parallel rankings across learners, indicating that our main conclusions are not artifacts of treating SUD transitions as purely binary outcomes. In the Strict stimulant specification, both models achieved higher discrimination and better precision–recall behavior, supporting the view that improved exposure definition (i.e., excluding likely therapeutic use without misuse codes) enhances predictive performance. EN’s robustness to multicollinearity and RF’s sensitivity to aggressive dimensionality reduction highlight the importance of preserving interpretable predictors when modeling complex, high-dimensional EHR datasets.

IPW-reweighted test-set metrics fell within the bootstrap confidence intervals of unweighted estimates across all model–cohort pairs, indicating that differences between EN and RF performance are unlikely to be driven by wearable participation. Overall, these results support combined use of interpretable linear models and flexible non-linear methods to triangulate conclusions about risk factors and to identify high-risk subgroups.

### 4.4. Clinical and public health implications

Our findings reinforce the importance of integrating SDoH into predictive frameworks and prevention strategies (Alegría et al., 2015). However, RF results indicate that among SDoH measures, income consistently dominated predictive importance, whereas other SDoH variables contributed primarily through linear associations captured by EN. Income, food insecurity, non-English language use, and related indicators contributed meaningfully to transition risk in EN models above and beyond demographics and behaviors, and their relative influence differed by substance type and modeling approach. This heterogeneity suggests that uniform screening thresholds may miss context-specific risks and underscores the need for flexible, contextually informed decision rules.

The distinct contributions of wearable-derived metrics between cannabis and stimulant transitions suggest different clinical applications. For cannabis, passive monitoring of activity and sleep may help identify individuals whose behavioral patterns, as reflected primarily in elastic net associations, signal elevated transition risk. For stimulants, structured assessment of sociodemographic disadvantage and cumulative stressors may be more informative than short-term behavioral sensing alone. Fairness profiles indicate that cannabis models may over-flag some minoritized groups, raising concerns about disproportionate intervention burdens; threshold selection and deployment strategies should therefore incorporate subgroup monitoring and safeguards.

The modest 1-year time-dependent AUCs in survival analyses highlight that, in low-incidence, administratively censored settings, models can rank-order risk reasonably well while still having limited short-horizon discrimination. This suggests that repeated, longitudinal risk assessment may be preferable to one-time screening for identifying individuals most likely to transition to SUD.

### 4.5. Limitations and future directions

Several limitations should be noted. Although All of Us is more diverse than many biomedical datasets, some populations remain underrepresented, potentially limiting generalizability. EHR-derived diagnoses likely undercapture true SUD prevalence and may reflect differential access, documentation, and stigma across groups. Observed associations are therefore correlational and may be influenced by selection mechanisms and residual confounding.

We lacked precise temporal information on substance use onset and escalation, limiting our ability to distinguish predictors of initiation from predictors of progression. Follow-up was constrained by available EHR records (median ~2 years among non-cases), and administrative censoring may underestimate later incident SUD. Survival models partially address this but remain limited by low early event rates and truncation.

Participants were assigned to a primary substance cohort based on earliest documented use, and polysubstance use was not jointly modeled. As a result, we cannot fully characterize trajectories involving co-use or multiple SUD outcomes. For stimulants, we could not fully separate therapeutic from non-medical use; our Strict specification mitigates some misclassification at the cost of reducing sample size.

Wearable measures were available only for a subset of participants and collapsed into person-level summaries, preventing evaluation of dynamic changes in behavior preceding transition. Several SDoH variables (e.g., housing instability, intimate partner violence, social isolation) had sparse reporting, limiting power to detect their effects. Future work should incorporate richer temporal modeling, more complete SDoH and policy-level data, and multilevel contextual measures (e.g., neighborhood disadvantage, state-level cannabis policies). Integrating genomic data, natural language processing of clinical notes, and high-frequency wearable streams could support adaptive, personalized prevention strategies.

Finally, fairness analyses indicated racial and ethnic disparities in predicted positive and error rates, particularly for cannabis. Because subgroup event counts were small, especially in stimulants, these estimates are exploratory. Nonetheless, they underscore the need for fairness-aware modeling, subgroup calibration checks, and incorporation of structural context to distinguish model bias from underlying disparities in exposure and access.

### 4.6. Summary

By integrating SDoH, wearable-derived behavioral metrics, and demographics in a large, diverse cohort, this study provides evidence that moderate prediction of SUD transitions among participants with cannabis and stimulant use is achievable, with substance-specific patterns in risk factors. Demographic and socioeconomic variables remain dominant predictors, while wearable measures contribute additional information primarily in elastic net models for cannabis transitions and more modestly for stimulant transitions. EN and RF showed comparable performance for cannabis and a non-significant RF advantage for stimulants, illustrating the complementary value of linear and non-linear approaches. Participation-bias checks and time-to-event sensitivity analyses support the robustness of these findings. Collectively, the results emphasize the need for multidimensional, equity-aware prevention strategies that account for both individual behavior and structural determinants when identifying individuals at risk for SUD transitions.

## Supplementary Material

MMC1

## Figures and Tables

**Fig. 1.**
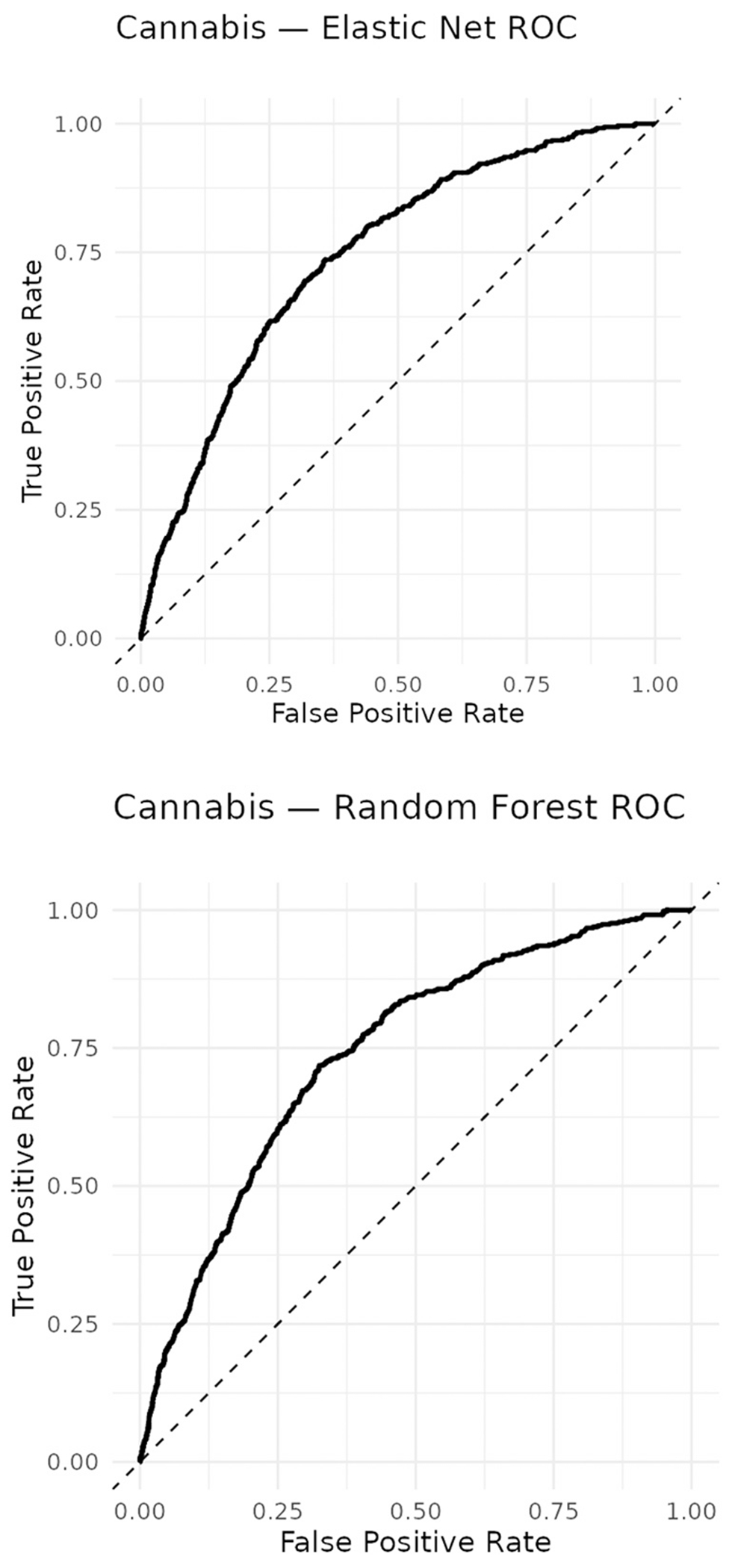
ROC Curves for Cannabis Models. Receiver operating characteristic (ROC) curves for elastic net and random forest cannabis models. The elastic net achieved an AUC of 0.740, while the random forest reached 0.741, demonstrating comparable performance.

**Fig. 2.**
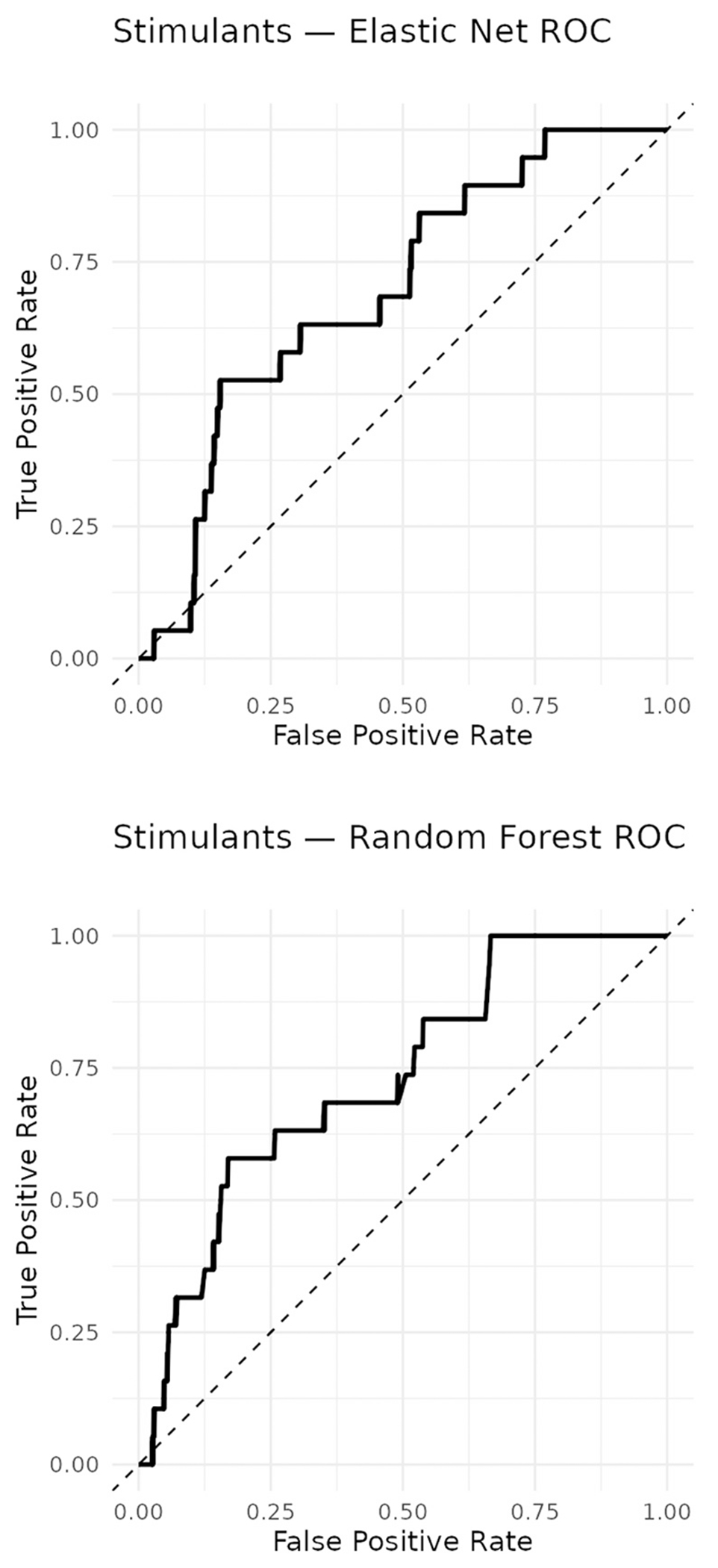
ROC Curves for Stimulant Models. ROC curves for stimulant models. Elastic net achieved an AUC of 0.698, and random forest achieved 0.732, highlighting stronger non-linear predictive performance in smaller stimulant cohorts, although the EN–RF difference was not statistically significant (DeLong’s p = 0.219).

**Table 1:** Demographic characteristics comparing participants with baseline cannabis use versus stimulant use.

Variable / Level	Cannabis (n = 142,730)	Stimulants (n = 4070)	Overall (n = 146,800)	Std Diff (S–C)	Overall SMD (multi-level)
***Age (years)*** †	48.6 (17.2) 49.0 [33.1, 63.5]	51.6 (16.8) 51.7 [38.1, 65.0]	48.6 (17.2) 49.1 [33.2, 63.6]	48.6 (17.2) 49.1 [33.2, 63.6]	—
***Sex at birth***					**0.276**
• *Female*	88,120 (61.7 %)	2869 (70.5 %)	90,989 (62.0 %)	0.180	
• *Male*	53,574 (37.5 %)	1145 (28.1 %)	54,719 (37.3 %)	−0.194	
• *Unknown*	1036 (0.7 %)	56 (1.4 %)	1092 (0.7 %)	0.076	
***Gender***					**0.278**
• *Female*	86,447 (60.6 %)	2856 (70.2 %)	89,303 (60.8 %)	0.197	
• *Male*	52,935 (37.1 %)	1125 (27.6 %)	54,060 (36.8 %)	−0.196	
• *Other/Unknown*	3348 (2.3 %)	89 (2.2 %)	3437 (2.3 %)	−0.011	
***Race***					**0.287**
• *Asian*	3606 (2.5 %)	114 (2.8 %)	3720 (2.5 %)	0.017	
• *Black or African American*	26,327 (18.4 %)	599 (14.7 %)	26,926 (18.3 %)	−0.096	
• *Multiple*	7697 (5.4 %)	166 (4.1 %)	7863 (5.4 %)	−0.058	
• *Other/Unknown*	20,307 (14.2 %)	937 (23.0 %)	21,244 (14.5 %)	0.250	
• *White*	84,793 (59.4 %)	2254 (55.4 %)	87,047 (59.3 %)	−0.082	
***Ethnicity***					**0.073**
• *Hispanic or Latino*	139,451 (97.7 %)	3945 (96.9 %)	143,396 (97.7 %)	−0.051	
• *Unknown*	3279 (2.3 %)	125 (3.1 %)	3404 (2.3 %)	0.051	

† Age reported as mean (SD) | median IQR

Values are presented as counts and percentages for categorical variables and means (SD) and medians [IQR] for continuous variables. Standardized mean differences (SMD) quantify effect sizes between stimulant and cannabis cohorts, with overall SMDs provided for multi-level categorical variables. We interpreted |SMD| < 0.10 as negligible, 0.10–0.20 as small, and > 0.20 as meaningful imbalance to contextualize participation differences.

**Table 2:** Candidate and selected variables by cohort and model.

Cohort	Elastic Net: Candidates	Elastic Net: Selected (non-zero)	Random Forest: Predictors Considered	Random Forest: Top-Ranked Predictors (reported)	Feature Families (EN Selected)	Feature Families (RF Top-Ranked)
**Cannabis**	33	26	35	15	Demographics (9), Fitbit Activity (7), Fitbit Sleep (6), SDoH (5), HR Zones (5), Age (1)	Demographics (3), Fitbit Activity (4), Fitbit Sleep (1), SDoH (4), HR Zones (2), Age (1)
**Stimulants**	31	9	35	15	Demographics (9), Fitbit Activity (7), Fitbit Sleep (6), SDoH (3), HR Zones (5), Age (1)	Demographics (4), Fitbit Activity (5), SDoH (3), HR Zones (2), Age (1)

After preprocessing, elastic net models included between 31 and 33 candidate features, of which 9–26 were retained with non-zero coefficients at the optimal penalty. Random forest models considered 35 predictors in both cohorts; for interpretability, we additionally report the top 15 random forest predictors ranked by variable importance. Elastic net–retained features spanned demographics, Fitbit-derived activity and sleep measures, heart-rate zones, and social determinants of health (SDoH). Top-ranked random forest predictors showed overlapping representation across these feature families, with cohort-specific differences in the relative prominence of activity, sleep, and SDoH domains.

## Data Availability

The data used in this work come from the All of Us Research Program Registered Tier dataset (version 8), accessible through the All of Us Researcher Workbench (https://www.researchallofus.org). Data are available only to qualified researchers who complete the required training and agree to abide by the All of Us Data and Statistics Dissemination Policy. Because of participant privacy protections, the dataset is not open to the public; however, approved researchers can gain access via the Workbench platform.

## Appendix A. Supporting information

Supplementary data associated with this article can be found in the online version at doi:10.1016/j.drugalcdep.2025.113012.
