# Supplementary material for: Comparing random forest and elastic net models to predict substance use disorder transitions in participants with cannabis and stimulant use: Evidence from the All of Us cohort
Source: Drug Alcohol Depend. Author manuscript; Available in PMC 2026 Jun 15. (PMC13266540; doi:10.1016/j.drugalcdep.2025.113012)
Supplement: MMC1 [file NIHMS2179762-supplement-MMC1.pdf]

Supplementary Material

Supplementary Table S1. Descriptive Statistics, Missingness, and Multicollinearity for Predictor Variables

(a) Continuous Predictor Variables

Domain	Variable	Label	N	Missing	Missing_pct	Mean	SD	Median	IQR	Min	Max	VIF_max	VIF_mean	High_VIF
Demographics	age_at_baseline	Age at baseline (years)	146800	0	0.00	48.65	17.18	49.10	30.30	18.0	88.7	1.15	1.15	No
Fitbit — Activity	n_days_act	Days with activity data	146800	138409	94.28	89.79	9.22	91.0	0.0	1.0	91.0	1.84	1.84	No
Fitbit — Activity	sedentary_min_mean	Sedentary minutes (mean / day)	146800	138409	94.28	892.14	77.63	884.93	0.0	0.0	1440.0	3.61	3.61	No
Fitbit — Activity	steps_cv	Steps (CV)	146800	139993	95.36	0.45	0.15	0.45	0.0	0.0	9.54	1.23	1.23	No
Fitbit — Activity	steps_mean	Steps (mean / day)	146800	138409	94.28	5965.34	1059.11	5976.04	0.0	0.0	67702.24	5.21	5.21	No
Fitbit — Activity	steps_sd	Steps (SD)	146800	138674	94.46	2676.27	455.50	2686.51	0.0	0.0	58388.5	2.66	2.66	No
Fitbit — Activity	very_active_min_mean	Very active minutes (mean / day)	146800	141190	96.18	8.29	5.59	7.91	0.0	0.0	447.91	2.78	2.78	No
Fitbit — Sleep & HR	hr_zone_days	Days with HR-zone data	146800	139530	95.05	46.96	7.65	46.0	0.0	1.0	91.0	1.34	1.34	No
Fitbit — Sleep & HR	n_days_sleep	Days with sleep data	146800	140583	95.76	72.23	7.50	73.0	0.0	1.0	91.0	2.00	2.00	No
Fitbit — Sleep & HR	prop_cardio	Time in Cardio zone	146800	0	0.00	0.00	0.01	0.00	0.0	0.0	0.57	2.29	2.29	No
Fitbit — Sleep & HR	prop_fat_burn	Time in Fat-burn zone	146800	0	0.00	0.04	0.04	0.03	0.0	0.0	0.97	2.33	2.33	No
Fitbit — Sleep & HR	prop_out_of_range	Time out of range	146800	0	0.00	0.87	0.09	0.88	0.0	0.0	1.0	3.29	3.29	No
Fitbit — Sleep & HR	prop_peak	Time in Peak zone	146800	0	0.00	0.00	0.00	0.00	0.0	0.0	0.33	1.57	1.57	No
Fitbit — Sleep & HR	sleep_eff_mean	Sleep efficiency (mean)	146800	140583	95.76	0.89	0.01	0.89	0.0	0.21	1.0	1.83	1.83	No
Fitbit — Sleep & HR	sleep_eff_sd	Sleep efficiency (SD)	146800	140830	95.93	0.04	0.01	0.04	0.0	0.0	0.57	3.00	3.00	No
Fitbit — Sleep & HR	sleep_min_mean	Sleep minutes (mean)	146800	140583	95.76	359.08	20.45	360.02	0.0	14.0	1133.0	1.50	1.50	No
Fitbit — Sleep & HR	sleep_min_sd	Sleep minutes (SD)	146800	140830	95.93	115.39	9.14	115.33	0.0	0.0	461.74	1.23	1.23	No
Fitbit — Sleep & HR	wake_after_wakeup_mean	Wake-after-wakeup (min; mean)	146800	140583	95.76	0.60	0.72	0.56	0.0	0.0	52.01	2.03	2.03	No

Supplementary Material

Domain	Variable	Label	N	Missing	Missing_pct	Mean	SD	Median	IQR	Min	Max	VIF_max	VIF_mean	High_VIF
Other	zero_step_day_prop	Zero-step days (prop.)	146800	0	0.00	0.01	0.10	0.00	0.0	0.0	1.0	4.57	4.57	No
Social Determinants of Health	sdoh_education_ord	Education (ordered)	146800	0	0.00	2.00	0.00	2.00	0.0	2.0	2.0			
Social Determinants of Health	sdoh_food_insec_bin	Food insecurity (any)	146800	0	0.00	0.04	0.19	0.00	0.0	0.0	1.0	3.79	3.79	No
Social Determinants of Health	sdoh_food_insec_ord	Food insecurity (severity)	146800	0	0.00	0.07	0.40	0.00	0.0	-2.0	15.0	3.73	3.73	No
Social Determinants of Health	sdoh_housing_instab_ord	Housing instability (severity)	146800	0	0.00	0.00	0.00	0.00	0.0	0.0	0.0			
Social Determinants of Health	sdoh_income_low	Low income (bin)	146800	0	0.00	0.86	0.35	1.00	0.0	0.0	1.0	1.04	1.04	No
Social Determinants of Health	sdoh_income_ord	Income (ordered)	146800	0	0.00	1.00	0.00	1.00	0.0	1.0	1.0			
Social Determinants of Health	sdoh_internet_access_bin	Internet access at home (yes/no)	146800	0	0.00	0.00	0.00	0.00	0.0	0.0	0.0			
Social Determinants of Health	sdoh_non_english_home	Non-English language at home	146800	0	0.00	0.02	0.12	0.00	0.0	0.0	1.0	1.04	1.04	No
Social Determinants of Health	sdoh_safety_ipv_bin	Safety / IPV (yes/no)	146800	0	0.00	0.00	0.00	0.00	0.0	0.0	0.0			
Social Determinants of Health	sdoh_social_iso_ord	Social isolation (severity)	146800	0	0.00	0.00	0.00	0.00	0.0	0.0	0.0			
Social Determinants of Health	sdoh_transport_prob_ord	Transportation problems (severity)	146800	0	0.00	0.10	0.41	0.00	0.0	0.0	4.0	1.08	1.08	No
Social Determinants of Health	sdoh_unemployed	Unemployed	146800	0	0.00	0.00	0.00	0.00	0.0	0.0	0.0			

Supplementary Material

Continuous predictor variables are grouped by domain. Statistics include N, missingness, mean, standard deviation (SD), median, interquartile range (IQR), minimum, and maximum. Variance inflation factor (VIF) values are reported as the maximum and mean across dummy-coded columns; High_VIF indicates VIF_max > 10.

(b) Categorical Predictor Variables

Domain	***Variable***	***Label***	***Level***	***Count***	***Percent***	***VIF_max***	***VIF_mean***	***High_VIF***
Cohort Indicator	substance_type	Cohort: (vs Cannabis)	Cannabis	142730.0	97.23			
Cohort Indicator	substance_type	Cohort: (vs Cannabis)	Stimulants	4070.0	2.77			
Demographics	ethnicity	Ethnicity:	Hispanic or Latino	143396.0	97.68			
Demographics	ethnicity	Ethnicity:	Unknown	3404.0	2.32			
Demographics	gender	Gender:	Female	89303.0	60.83			
Demographics	gender	Gender:	Male	54060.0	36.83			
Demographics	gender	Gender:	Other/Unknown	3437.0	2.34			
Demographics	race	Race:	White	87047.0	59.3			
Demographics	race	Race:	Black or African American	26926.0	18.34			
Demographics	race	Race:	Asian	3720.0	2.53			
Demographics	race	Race:	Multiple	7863.0	5.36			
Demographics	race	Race:	Other/Unknown	21244.0	14.47			
Demographics	sex_at_birth	Sex at birth:	Female	90989.0	61.98	37.33	9.21	Yes
Demographics	sex_at_birth	Sex at birth:	Male	54719.0	37.27	37.33	9.21	Yes
Demographics	sex_at_birth	Sex at birth:	Unknown	1092.0	0.74	37.33	9.21	Yes

Categorical predictor variables are grouped by domain and summarized by level, with counts and percentages reported for each. Variance inflation factor (VIF) values are reported as the maximum and mean across dummy-coded columns; High_VIF indicates VIF_max > 10.

Supplementary Table S2. With-Fitbit vs No-Fitbit Participation (Demographic Characteristics with SMDs)

**Cohort**	**Variable**	**Type**	**No Fitbit (mean±sd)**	**With Fitbit (mean±sd)**	**SMD**

Supplementary Material

Overall	age_at_baseline	Continuous	48.47 ± 17.19	50.87 ± 16.1	0.144
Overall	steps_mean	Continuous	4874.81 ± 4425.66	6142.15 ± 4401.63	0.287
Overall	steps_sd	Continuous	2229.34 ± 2193.56	2678.55 ± 1903.73	0.219
Overall	steps_cv	Continuous	0.61 ± 0.43	0.56 ± 0.68	-0.089
Overall	sedentary_min_mean	Continuous	866.16 ± 478.49	570.77 ± 453.92	-0.633
Overall	very_active_min_mean	Continuous	16.18 ± 24.49	19.51 ± 22.74	0.141
Overall	sleep_min_mean	Continuous	349.18 ± 132.63	271.46 ± 162.17	-0.525
Overall	sleep_min_sd	Continuous	112.4 ± 74.42	91.58 ± 59.65	-0.309
Overall	sleep_eff_mean	Continuous	0.87 ± 0.13	0.7 ± 0.36	-0.614
Overall	sleep_eff_sd	Continuous	0.05 ± 0.06	0.04 ± 0.04	-0.177
Overall	wake_after_wakeup_mean	Continuous	6.32 ± 37.58	71.27 ± 142.99	0.621
Overall	hr_zone_days	Continuous	4.58 ± 3.61	58.57 ± 27.78	2.726
Overall	n_days_act	Continuous	4.65 ± 3.75	75.99 ± 26.95	3.707
Overall	n_days_sleep	Continuous	3.53 ± 3.22	61.16 ± 31.28	2.592
Overall	zero_step_day_prop	Continuous	0.2 ± 0.4	0.19 ± 0.38	-0.035
Overall	sex_at_birth	Categorical	Female: 93.8 | 6.2 || Male: 96.3 | 3.7 || Unknown: 97.2 | 2.8 || Unknown/NA: NaN | NaN		0.256
Overall	gender	Categorical	Female: 93.8 | 6.2 || Male: 96.3 | 3.7 || Other/Unknown: 93.5 | 6.5 || Unknown/NA: NaN | NaN		0.248
Overall	race	Categorical	White: 93.1 | 6.9 || Black or African American: 98.2 | 1.8 || Asian: 94.3 | 5.7 || Multiple: 94.1 | 5.9 || Other/Unknown: 97.4 | 2.6 || Unknown/NA: NaN | NaN		0.488
Overall	ethnicity	Categorical	Not Hispanic or Latino: NaN | NaN || Hispanic or Latino: 94.7 | 5.3 || Unknown: 96.3 | 3.7 || Unknown/NA: NaN | NaN		0.053
Overall	sdoh_income_low	Categorical	0: 98.2 | 1.8 || 1: 94.2 | 5.8 || Unknown/NA: 77.8 | 22.2		0.328
Overall	sdoh_non_english_home	Categorical	0: 91.8 | 8.2 || 1: 95.0 | 5.0 || Unknown/NA: 95.5 | 4.5		0.268
Overall	sdoh_food_insec_bin	Categorical	0: 91.9 | 8.1 || 1: 93.7 | 6.3 || Unknown/NA: 95.5 | 4.5		0.259
Cannabis	age_at_baseline	Continuous	48.39 ± 17.2	50.82 ± 16.09	0.146
Cannabis	steps_mean	Continuous	4902.93 ± 4424.69	6156.78 ± 4413.61	0.284
Cannabis	steps_sd	Continuous	2238.09 ± 2194.76	2683.57 ± 1907.37	0.217
Cannabis	steps_cv	Continuous	0.61 ± 0.42	0.56 ± 0.69	-0.075
Cannabis	sedentary_min_mean	Continuous	865.31 ± 475.49	569.71 ± 454.18	-0.636

Supplementary Material

Cannabis	very_active_min_mean	Continuous	16.29 ± 24.6	19.63 ± 22.79	0.141
Cannabis	sleep_min_mean	Continuous	349.79 ± 131.89	271.15 ± 162.33	-0.532
Cannabis	sleep_min_sd	Continuous	112.37 ± 73.65	91.33 ± 59.71	-0.314
Cannabis	sleep_eff_mean	Continuous	0.87 ± 0.12	0.7 ± 0.36	-0.631
Cannabis	sleep_eff_sd	Continuous	0.05 ± 0.06	0.04 ± 0.04	-0.191
Cannabis	wake_after_wakeup_mean	Continuous	5.25 ± 31.44	71.89 ± 143.62	0.641
Cannabis	hr_zone_days	Continuous	4.57 ± 3.62	58.49 ± 27.78	2.722
Cannabis	n_days_act	Continuous	4.67 ± 3.76	75.99 ± 26.93	3.709
Cannabis	n_days_sleep	Continuous	3.53 ± 3.23	61.2 ± 31.26	2.595
Cannabis	zero_step_day_prop	Continuous	0.2 ± 0.4	0.19 ± 0.38	-0.034
Cannabis	sex_at_birth	Categorical	Female: 93.7 | 6.3 || Male: 96.3 | 3.7 || Unknown: 97.2 | 2.8 || Unknown/NA: NaN | NaN		0.257
Cannabis	gender	Categorical	Female: 93.8 | 6.2 || Male: 96.3 | 3.7 || Other/Unknown: 93.4 | 6.6 || Unknown/NA: NaN | NaN		0.25
Cannabis	race	Categorical	White: 93.0 | 7.0 || Black or African American: 98.2 | 1.8 || Asian: 94.2 | 5.8 || Multiple: 94.0 | 6.0 || Other/Unknown: 97.4 | 2.6 || Unknown/NA: NaN | NaN		0.49
Cannabis	ethnicity	Categorical	Not Hispanic or Latino: NaN | NaN || Hispanic or Latino: 94.7 | 5.3 || Unknown: 96.3 | 3.7 || Unknown/NA: NaN | NaN		0.052
Cannabis	sdoh_income_low	Categorical	0: 98.2 | 1.8 || 1: 94.1 | 5.9 || Unknown/NA: 77.8 | 22.2		0.328
Cannabis	sdoh_non_english_home	Categorical	0: 91.8 | 8.2 || 1: 95.0 | 5.0 || Unknown/NA: 95.4 | 4.6		0.265
Cannabis	sdoh_food_insec_bin	Categorical	0: 91.9 | 8.1 || 1: 93.7 | 6.3 || Unknown/NA: 95.4 | 4.6		0.254
Stimulants	age_at_baseline	Continuous	51.38 ± 16.7	53.15 ± 16.71	0.106
Stimulants	steps_mean	Continuous	3835.62 ± 4454.14	5493.65 ± 3788.26	0.401
Stimulants	steps_sd	Continuous	1882.37 ± 2210.76	2457.34 ± 1726.11	0.29
Stimulants	steps_cv	Continuous	0.92 ± 0.55	0.51 ± 0.28	-0.944
Stimulants	sedentary_min_mean	Continuous	897.6 ± 593.29	617.85 ± 440.82	-0.535
Stimulants	very_active_min_mean	Continuous	11.64 ± 19.31	14.62 ± 19.92	0.152
Stimulants	sleep_min_mean	Continuous	296.25 ± 205.65	284.57 ± 155.33	-0.064
Stimulants	sleep_min_sd	Continuous	114.18 ± 142.72	102.47 ± 56.24	-0.108
Stimulants	sleep_eff_mean	Continuous	0.66 ± 0.44	0.75 ± 0.32	0.246
Stimulants	sleep_eff_sd	Continuous	0.02 ± 0.02	0.05 ± 0.05	0.864

Supplementary Material

Stimulants	wake_after_wakeup_mean	Continuous	98.8 ± 197.6	44.6 ± 109.96	-0.339
Stimulants	hr_zone_days	Continuous	5.25 ± 2.87	62.18 ± 27.53	2.909
Stimulants	n_days_act	Continuous	4 ± 3.37	75.6 ± 27.96	3.596
Stimulants	n_days_sleep	Continuous	3.25 ± 2.06	59.43 ± 32.15	2.466
Stimulants	zero_step_day_prop	Continuous	0.22 ± 0.42	0.19 ± 0.38	-0.078
Stimulants	sex_at_birth	Categorical	Female: 95.2 | 4.8 || Male: 97.2 | 2.8 || Unknown: 98.2 | 1.8 || Unknown/NA: NaN | NaN		0.241
Stimulants	gender	Categorical	Female: 95.3 | 4.7 || Male: 97.1 | 2.9 || Other/Unknown: 97.8 | 2.2 || Unknown/NA: NaN | NaN		0.232
Stimulants	race	Categorical	White: 94.4 | 5.6 || Black or African American: 97.8 | 2.2 || Asian: 96.5 | 3.5 || Multiple: 96.4 | 3.6 || Other/Unknown: 97.9 | 2.1 || Unknown/NA: NaN | NaN		0.436
Stimulants	ethnicity	Categorical	Not Hispanic or Latino: NaN | NaN || Hispanic or Latino: 95.8 | 4.2 || Unknown: 97.5 | 2.5 || Unknown/NA: NaN | NaN		0.084
Stimulants	sdoh_income_low	Categorical	0: 98.3 | 1.7 || 1: 95.3 | 4.7 || Unknown/NA: NaN | NaN		0.323
Stimulants	sdoh_non_english_home	Categorical	0: 91.9 | 8.1 || 1: NaN | NaN || Unknown/NA: 96.5 | 3.5		0.361
Stimulants	sdoh_food_insec_bin	Categorical	0: 91.5 | 8.5 || 1: 93.0 | 7.0 || Unknown/NA: 96.8 | 3.2		0.435

Rows summarize overall, cannabis, and stimulant cohorts; continuous variables are reported as mean ± SD and categorical entries show "No-Fitbit % | With-Fitbit %". SMDs quantify imbalance between groups (absolute SMD ≈0.1 small, ≈0.2–0.3 modest, ≥0.5 large); sign indicates direction, but interpretation is by magnitude. As expected, participation-dependent wearables counters (e.g., n_days_act, n_days_sleep, hr_zone_days) show very large SMDs reflecting data availability rather than baseline differences. Outside those, age is modestly higher among With-Fitbit participants (Overall SMD = 0.144), and several activity/sleep summaries show small-to-moderate differences (e.g., steps_mean SMD ≈ 0.29; sedentary_min_mean SMD ≈ –0.63). Demographic/SDoH imbalances are generally small-to-moderate (e.g., race SMD ≈ 0.49 overall), consistent across cannabis and stimulant strata. "Unknown/NA" reflects missing survey responses; SMDs were computed using pooled SD for continuous variables and a generalized SMD for multi-level categorical variables. Missingness in survey-based SDoH variables reflected standard non-response patterns in All of Us and did not differ substantially between wearable participants and non-participants. These variables were included in the IPTW model, mitigating imbalance due to missingness patterns.

Supplementary Table S3. Expanded Classification Metrics for Each Model and Cohort

Cohort	Model	Threshold	Threshold_Label	AUC	Sensitivity	Specificity	PPV	NPV	F1	Accuracy	Balanced_Accuracy	Brier	Tjur_R2	Null_Information_Rate	McFadden_R2	Nagelkerke_R2
Cannabis	Elastic Net	0.500	0.50	0.740	0.687	0.676	0.034	0.992	0.064	0.677	0.682	0.209	0.167	0.984	-6.332	-12.114
Cannabis	Elastic Net	0.480	YoudenJ	0.740	0.737	0.648	0.033	0.993	0.063	0.649	0.692	0.209	0.167	0.984	-6.332	-12.114

Supplementary Material

Cohort	Model	Threshold	Threshold_Label	AUC	Sensitivity	Specificity	PPV	NPV	F1	Accuracy	Balanced_Accuracy	Brier	Tjur_R2	Null_Information_Rate	McFadden_R2	Nagelkerke_R2
Cannabis	Random Forest	0.500	0.50	0.741	0.000	1.000		0.984		0.984	0.500	0.016	0.012	0.984	0.070	0.075
Cannabis	Random Forest	0.019	YoudenJ	0.741	0.720	0.667	0.034	0.993	0.065	0.668	0.693	0.016	0.012	0.984	0.070	0.075
Stimulants	Elastic Net	0.500	0.50	0.698	0.632	0.680	0.045	0.987	0.084	0.679	0.656	0.188	0.128	0.977	-4.046	-7.296
Stimulants	Elastic Net	0.622	YoudenJ	0.698	0.526	0.852	0.078	0.987	0.136	0.844	0.689	0.188	0.128	0.977	-4.046	-7.296
Stimulants	Random Forest	0.500	0.50	0.732	0.000	1.000		0.977		0.977	0.500	0.022	0.023	0.977	0.074	0.082
Stimulants	Random Forest	0.049	YoudenJ	0.732	0.579	0.851	0.085	0.988	0.148	0.844	0.715	0.022	0.023	0.977	0.074	0.082

Metrics are reported on the independent test sets for elastic net and random forest (Cannabis, Stimulants). For each model we report AUC and threshold-dependent metrics at a fixed threshold of 0.50 and at the Youden J index threshold. Reported metrics include sensitivity, specificity, PPV, NPV, F1 score, accuracy, balanced accuracy, Brier score, Tjur's R^2, null information rate, and pseudo-R^2 (McFadden, Nagelkerke).
Note: F1 scores appear small because the positive predictive value (PPV) is very low due to the rare outcome prevalence (≈1.6% cannabis SUD, ≈2.3% stimulant SUD); this is expected behavior when prevalence is low, even with reasonable sensitivity.

Supplementary Table S4. IPW-reweighted Performance on Independent Test Sets.

Cohort	Model	AUC_est	AUC_L	AUC_U	PRAUC_est	PRAUC_L	PRAUC_U
Cannabis	Elastic Net	0.742	0.719	0.764	0.044	0.037	0.053
Cannabis	Random Forest	0.74	0.716	0.763	0.043	0.036	0.051
Stimulants	Elastic Net	0.69	0.579	0.792	0.041	0.021	0.069
Stimulants	Random Forest	0.725	0.608	0.825	0.054	0.026	0.095

IPW was estimated from baseline demographics and SDoH to align the test-set distribution with the target population (wearable participation propensity accounted for), and metrics were recomputed under these weights. Entries report AUC and PR-AUC point estimates with 95% bootstrap CIs (AUC_L–AUC_U; PRAUC_L–PRAUC_U). As expected in a low-prevalence setting (~1.6% cannabis SUD; ~2.3% stimulant SUD), PR-AUC values are small; reweighting preserves the pattern of moderate AUCs in cannabis (elastic net ~0.74; random forest ~0.74) and higher random forest discrimination in stimulants (AUC ~0.73) with overlapping confidence intervals.

Supplementary Material

Supplementary Table S5. Elastic Net Regression Coefficients for All Predictors

Cohort	Family	Label	feature	coefficient
Cannabis	Other	Zero-step days (prop.)	zero_step_day_prop	-1.86933
Cannabis	Demographics (one-hot)	Race: Black or African American	raceBlack or African American	0.96516
Cannabis	Demographics (one-hot)	Race: Asian	raceAsian	-0.93211
Cannabis	Demographics (one-hot)	Race: Other/Unknown	raceOther/Unknown	0.84379
Cannabis	Demographics (one-hot)	Sex at birth: Unknown	sex_at_birthUnknown	0.73449
Cannabis	SDoH	Low income (bin)	sdoh_income_low	-0.70136
Cannabis	Demographics (one-hot)	Race: Multiple	raceMultiple	0.62813
Cannabis	SDoH	Food insecurity (any)	sdoh_food_insec_bin	-0.41659
Cannabis	SDoH	Non-English language at home	sdoh_non_english_home	-0.31223
Cannabis	HR Zones	Time in Fat-burn zone	prop_fat_burn	-0.26158
Cannabis	SDoH	Transportation problems (severity)	sdoh_transport_prob_ord	-0.24423
Cannabis	HR Zones	Time out of range	prop_out_of_range	0.21314
Cannabis	Demographics (one-hot)	Sex at birth: Male	sex_at_birthMale	0.21279
Cannabis	Demographics (one-hot)	Gender: Male	genderMale	0.17625
Cannabis	Fitbit Activity	Steps (CV)	steps_cv	-0.15631
Cannabis	Fitbit Sleep	Wake-after-wakeup (min; mean)	wake_after_wakeup_mean	-0.11240
Cannabis	Demographics (one-hot)	Ethnicity: Unknown	ethnicityUnknown	0.07404
Cannabis	Demographics (one-hot)	Gender: Other/Unknown	genderOther/Unknown	0.04519
Cannabis	Age	Age at baseline (years)	age_at_baseline	-0.02627
Cannabis	Fitbit Activity	Very active minutes (mean / day)	very_active_min_mean	-0.02096
Cannabis	SDoH	Food insecurity (severity)	sdoh_food_insec_ord	0.01046
Cannabis	Fitbit Activity	Days with activity data	n_days_act	0.01021
Cannabis	Fitbit Sleep	Days with sleep data	n_days_sleep	0.00640
Cannabis	HR Zones	Days with HR-zone data	hr_zone_days	-0.00378

Supplementary Material

Cohort	Family	Label	feature	coefficient
Cannabis	Fitbit Sleep	Sleep minutes (mean)	sleep_min_mean	-0.00206
Cannabis	Fitbit Sleep	Sleep minutes (SD)	sleep_min_sd	-0.00137
Cannabis	Fitbit Activity	Steps (mean / day)	steps_mean	-0.00011
Cannabis	Fitbit Activity	Sedentary minutes (mean / day)	sedentary_min_mean	0.00000
Cannabis	Fitbit Sleep	Sleep efficiency (SD)	sleep_eff_sd	0.00000
Cannabis	Fitbit Sleep	Sleep efficiency (mean)	sleep_eff_mean	0.00000
Cannabis	Fitbit Activity	Steps (SD)	steps_sd	0.00000
Cannabis	HR Zones	Time in Cardio zone	prop_cardio	0.00000
Cannabis	HR Zones	Time in Peak zone	prop_peak	0.00000
Stimulants	Demographics (one-hot)	Race: Asian	raceAsian	-1.65024
Stimulants	SDoH	Food insecurity (any)	sdoh_food_insec_bin	-1.42414
Stimulants	SDoH	Low income (bin)	sdoh_income_low	-1.15334
Stimulants	Demographics (one-hot)	Sex at birth: Male	sex_at_birthMale	1.04833
Stimulants	Demographics (one-hot)	Gender: Male	genderMale	0.50892
Stimulants	Fitbit Activity	Sedentary minutes (mean / day)	sedentary_min_mean	0.45067
Stimulants	Demographics (one-hot)	Race: Black or African American	raceBlack or African American	0.36016
Stimulants	HR Zones	Time in Peak zone	prop_peak	0.34494
Stimulants	Fitbit Activity	Steps (mean / day)	steps_mean	0.24079
Stimulants	Fitbit Sleep	Sleep efficiency (mean)	sleep_eff_mean	0.23544
Stimulants	Fitbit Sleep	Days with sleep data	n_days_sleep	0.21142
Stimulants	Fitbit Activity	Steps (SD)	steps_sd	0.20174
Stimulants	Fitbit Sleep	Sleep efficiency (SD)	sleep_eff_sd	0.16994
Stimulants	HR Zones	Time in Cardio zone	prop_cardio	0.10649
Stimulants	Fitbit Activity	Days with activity data	n_days_act	0.08004
Stimulants	Demographics (one-hot)	Gender: Other/Unknown	genderOther/Unknown	0.07276
Stimulants	Demographics (one-hot)	Race: Other/Unknown	raceOther/Unknown	-0.04256
Stimulants	Fitbit Activity	Very active minutes (mean / day)	very_active_min_mean	0.03881

Supplementary Material

Cohort	Family	Label	feature	coefficient
Stimulants	HR Zones	Time in Fat-burn zone	prop_fat_burn	-0.03032
Stimulants	Fitbit Sleep	Sleep minutes (SD)	sleep_min_sd	-0.01895
Stimulants	Fitbit Sleep	Sleep minutes (mean)	sleep_min_mean	-0.01387
Stimulants	HR Zones	Time out of range	prop_out_of_range	0.00924
Stimulants	Fitbit Sleep	Wake-after-wakeup (min; mean)	wake_after_wakeup_mean	0.00636
Stimulants	HR Zones	Days with HR-zone data	hr_zone_days	0.00421
Stimulants	Age	Age at baseline (years)	age_at_baseline	-0.00260
Stimulants	Demographics (one-hot)	Ethnicity: Unknown	ethnicityUnknown	0.00000

Coefficients (log-odds scale) are reported for all predictors included in the elastic net models for cannabis and stimulant cohorts. Positive coefficients indicate higher predicted risk of subsequent SUD diagnosis, while negative coefficients indicate lower risk. Zero coefficients indicate predictors that were penalized to zero during model fitting.

Supplementary Cannabis Table S6. Random-Forest permutation importances (Cannabis cohort)
Top 15 predictors ranked by permutation importance

Feature	Permutation importance	p-value
Sex at birth	1.32e-04	0.005
Proportion time out of range (HR)	1.20e-04	1.000
Race	1.18e-04	0.005
Age at baseline	1.14e-04	0.005
Proportion time fat-burn (HR)	1.02e-04	1.000
Gender	9.89e-05	0.005
Mean daily sedentary minutes	7.56e-05	0.891
Low income (SDoH)	6.17e-05	0.005
Mean nightly sleep minutes	5.45e-05	0.990
Sleep duration SD	5.03e-05	0.960
Mean daily steps	4.62e-05	1.000
Proportion time cardio (HR)	4.36e-05	0.995
Steps SD	4.23e-05	0.995

Supplementary Material

Proportion time peak (HR)	3.41e-05	0.975
Steps CV	3.38e-05	0.980

Permutation-tested random-forest importances (B = 200 permutations) from the tuned Cannabis model (mtry = 5, min.node.size = 50). Importance is defined as the mean decrease in held-out AUC following feature permutation. The top 15 predictors are shown. Two-sided permutation p-values are reported; significance is not used as a selection criterion for ranking. Permutation p-values may equal 1.000 when the observed AUC decrease is not greater than any decrease seen under permutation (i.e., the feature provides no measurable out-of-sample contribution beyond noise in this evaluation).

Supplementary PDP Table S7. Mean Partial Dependence Summary — Cannabis Cohort

Feature	Random Forest Importance (Scaled)	Approximate Change-Point (PDP)	Interpretation of Change-Point	Elastic Net Direction (Coefficient)
Age at baseline	1437.0	≈ 40 years	Risk increases steeply through early adulthood and levels off after mid-life	−0.0274 (higher age → lower odds in adjusted linear model)
Low income	254.0	≈ 0.53 probability / midrange of distribution	Threshold-like elevation in risk once participants exceed moderate likelihood of low income	−0.656 (lower income associated with increased risk)

Mean Partial Dependence estimates illustrate non-linear relationships between age and socioeconomic disadvantage and subsequent cannabis-related SUD transition risk. Change-points represent approximate inflection regions derived from aggregated PDP patterns. ICE curves were used only to verify direction and location of change-points but are not displayed due to All of Us N<20 privacy restrictions.

Supplementary Stimulant Table S8. Random-Forest Permutation Importances (Stimulants cohort)

Top 15 predictors ranked by permutation importance

Feature	Permutation importance	p-value
Sex at birth	1.29e-03	0.010
Gender	1.06e-03	0.020
Low income (SDoH)	7.58e-04	0.005
Age at baseline	5.79e-04	0.090
Race	1.35e-04	0.428
Ethnicity	8.17e-05	0.119
Food insecurity (binary)	3.34e-05	0.284

Supplementary Material

Feature	Permutation importance	p-value
Food insecurity (ordinal)	2.48e-05	0.358
Mean daily sedentary minutes	6.89e-06	0.667
Number of active days	6.82e-06	0.488
Proportion time fat-burn (HR)	5.66e-06	0.821
Mean daily steps	3.19e-06	0.692
Number of sleep days	3.37e-07	0.493
HR zone days	3.05e-07	0.567
Wake after wake-up (mean)	1.46e-07	0.577

Permutation-tested random-forest importances (B = 200 permutations) from the tuned Stimulants model (mtry = 7, min.node.size = 5). Importance reflects the mean decrease in held-out AUC following permutation. The top 15 predictors are reported. Two-sided permutation p-values are shown for inference but are not used to define ranking. Permutation p-values may equal 1.000 when the observed AUC decrease is not greater than any decrease seen under permutation (i.e., the feature provides no measurable out-of-sample contribution beyond noise in this evaluation).

Supplementary PDP Table S9. Mean Partial Dependence Summary — Stimulants Cohort

Feature	Random Forest Importance (Scaled)	Approximate Change-Point (PDP)	Interpretation of Change-Point	Elastic Net Direction (Coefficient)
Age at baseline	114.0	≈ 46–47 years	Risk remains moderate until mid-forties, then increases	−0.0136 (higher age → lower odds in linear model)
Low income	49.9	Step change at any presence of low-income indicator (≈ 0)	Risk elevates in a *stepwise* (not gradient) manner when low income is present	−1.12 (lower income strongly associated with increased risk)

Mean Partial Dependence estimates for the stimulant cohort indicate threshold-like effects of socioeconomic disadvantage and a late-onset inflection in age-related risk. ICE-based heterogeneity summaries are reported descriptively but not visualized to comply with All of Us data display requirements.

Supplementary Material

Supplementary Table S10. Fairness Metrics by Race (with 95% CIs)

Cohort	Model	Subgroup	N	Events	Non-Events	PPR	PPR_low	PPR_high	FPR	FPR_low	FPR_high	FNR	FNR_low	FNR_high
Cannabis	Elastic Net	White	16965	129	16836	0.065	0.06138787	0.06880909	0.064	0.06040142	0.06779751	0.783	0.70424284	0.84538921
Cannabis	Elastic Net	White	16965	129	16836	0.094	0.08970001	0.09848382	0.092	0.08772668	0.09645947	0.713	0.62966783	0.78401282
Cannabis	Elastic Net	Black or African American	5272	178	5094	0.821	0.81041915	0.83111338	0.817	0.80614393	0.8273783	0.079	0.04769188	0.12809628
Cannabis	Elastic Net	Black or African American	5272	178	5094	0.858	0.84831686	0.86716178	0.855	0.84506315	0.86440181	0.051	0.02713208	0.09383914
Cannabis	Elastic Net	Asian	726	3	723	0.006	0.00242339	0.01477706	0.006	0.00241912	0.01480279	1	0.43849392	1
Cannabis	Elastic Net	Asian	726	3	723	0.007	0.00301308	0.01617684	0.007	0.0030081	0.01620323	1	0.43849392	1
Cannabis	Elastic Net	Multiple	1549	36	1513	0.59	0.56531329	0.6142414	0.587	0.5619999	0.61155943	0.278	0.15865194	0.44015935
Cannabis	Elastic Net	Multiple	1549	36	1513	0.644	0.61982555	0.66746196	0.64	0.61548671	0.66380415	0.222	0.11700195	0.38060859
Cannabis	Elastic Net	Other/Unknown	3999	116	3883	0.766	0.75262645	0.77886298	0.763	0.74936868	0.77611144	0.112	0.06662046	0.18225472
Cannabis	Elastic Net	Other/Unknown	3999	116	3883	0.802	0.78936175	0.81405858	0.799	0.78610222	0.81130674	0.095	0.05390361	0.16206147
Cannabis	Random Forest	White	16965	129	16836	0	0	0.00022639	0	1.36E-20	0.00022813	1	0.97108135	1
Cannabis	Random Forest	White	16965	129	16836	0.05	0.04682103	0.05338273	0.048	0.04487278	0.05133345	0.76	0.67946543	0.82549687
Cannabis	Random Forest	Black or African American	5272	178	5094	0	0	0.00072815	0	0	0.00075357	1	0.97887392	1
Cannabis	Random Forest	Black or African American	5272	178	5094	0.782	0.77065132	0.79293801	0.777	0.76536252	0.78822	0.084	0.05152391	0.13405298
Cannabis	Random Forest	Asian	726	3	723	0	0	0.00526361	0	0	0.00528533	1	0.43849392	1
Cannabis	Random Forest	Asian	726	3	723	0.357	0.32298442	0.39252097	0.354	0.31999715	0.38954617	0	0	0.56150608
Cannabis	Random Forest	Multiple	1549	36	1513	0	0	0.00247392	0	0	0.00253263	1	0.90357817	1
Cannabis	Random Forest	Multiple	1549	36	1513	0.68	0.65634861	0.70276078	0.677	0.65301422	0.70008922	0.167	0.07892407	0.31929287
Cannabis	Random Forest	Other/Unknown	3999	116	3883	0	5.42E-20	0.00095972	0	0	0.00098836	1	0.96794435	1
Cannabis	Random Forest	Other/Unknown	3999	116	3883	0.792	0.77914285	0.80429667	0.789	0.77588382	0.80154491	0.095	0.05390361	0.16206147
Stimulants	Elastic Net	White	440	7	433	0.273	0.23347146	0.31645807	0.27	0.23034056	0.3137047	0.571	0.2501539	0.84152998
Stimulants	Elastic Net	White	440	7	433	0.111	0.08494863	0.14378523	0.106	0.08038972	0.13853998	0.571	0.2501539	0.84152998
Stimulants	Elastic Net	Black or African American	104	3	101	0.529	0.43375037	0.62218352	0.535	0.43823327	0.62920179	0.667	0.20783859	0.93861838
Stimulants	Elastic Net	Black or African American	104	3	101	0.24	0.16812405	0.33039971	0.238	0.16551852	0.32968186	0.667	0.20783859	0.93861838

Supplementary Material

Stimulants	Elastic Net	Other/Unknown	209	9	200	0.383	0.3197691	0.45045439	0.36	0.29669088	0.428586	0.111	0.01985616	0.43488468
Stimulants	Elastic Net	Other/Unknown	209	9	200	0.234	0.18172003	0.29588209	0.215	0.16371793	0.2770243	0.333	0.12039238	0.64552463
Stimulants	Random Forest	White	440	7	433	0	0	0.00865534	0	0	0.00879403	1	0.64566116	1
Stimulants	Random Forest	White	440	7	433	0.116	0.089347	0.14930031	0.111	0.0847647	0.14407706	0.571	0.2501539	0.84152998
Stimulants	Random Forest	Black or African American	104	3	101	0	3.47E-18	0.03562262	0	0	0.03664194	1	0.43849392	1
Stimulants	Random Forest	Black or African American	104	3	101	0.269	0.19313061	0.36132704	0.267	0.19042517	0.36064998	0.667	0.20783859	0.93861838
Stimulants	Random Forest	Other/Unknown	209	9	200	0	0	0.0180491	0	0	0.01884601	1	0.70084725	1
Stimulants	Random Forest	Other/Unknown	209	9	200	0.258	0.20342447	0.3213113	0.235	0.18157345	0.29841493	0.222	0.0631215	0.54720743

Predicted-positive rate (PPR), false-positive rate (FPR), and false-negative rate (FNR) are reported for each racial subgroup within cohort and model. N is the subgroup size in the test set; Events/Non-Events are the numbers with/without the outcome. Each subgroup appears twice because metrics are shown at two operating points: the validation-selected threshold (Youden J) and an alternative operating point used in sensitivity analyses (fixed top-k screening rate). Confidence intervals reflect binomial uncertainty (Wilson) with fold-wise aggregation; when subgroup event counts are extremely small, bounds may reach 0 or 1. These estimates are diagnostic and should be interpreted cautiously given the low prevalence and small subgroup event counts.

Multicollinearity & Sensitivity Analyses

Supplementary Material

Supplementary Figure S1. Pairwise Correlation Heatmaps of Predictor Variables

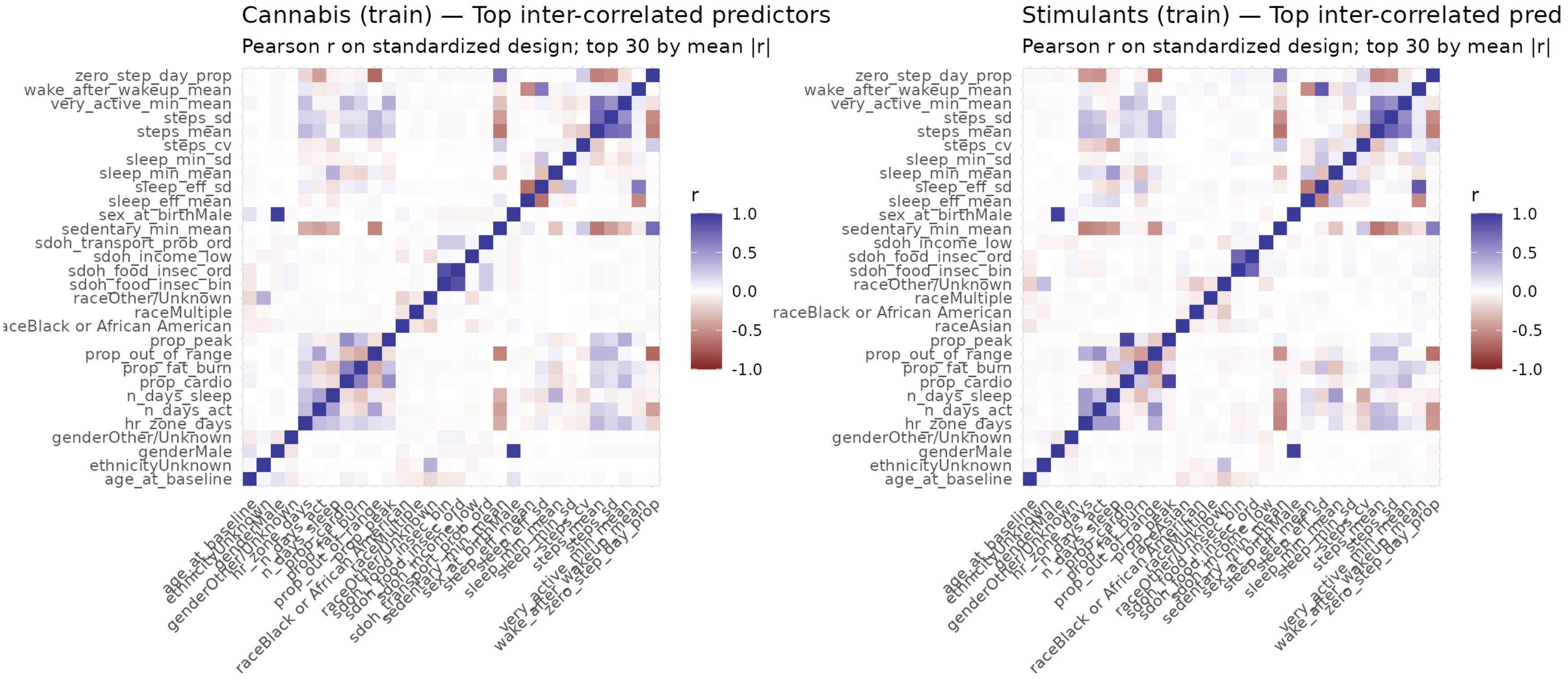

Heatmaps display pairwise Pearson correlation coefficients among predictors separately for the Cannabis cohort (left) and the Stimulant cohort (right). Blue tones indicate positive correlations and red tones indicate negative correlations, with intensity proportional to |r|. Most predictors exhibit only modest correlations, though several clusters of strong correlation (|r| ≥ 0.90) are apparent, particularly within related wearable-derived metrics (e.g., steps_mean vs. steps_sd, sleep_min_mean vs. sleep_min_sd). These clusters were used to guide the correlation-pruned sensitivity analyses (Supplementary Figure S3).

Supplementary Material

Supplementary Figure S2. Variance Inflation Factor (VIF) Distributions Across Cohorts

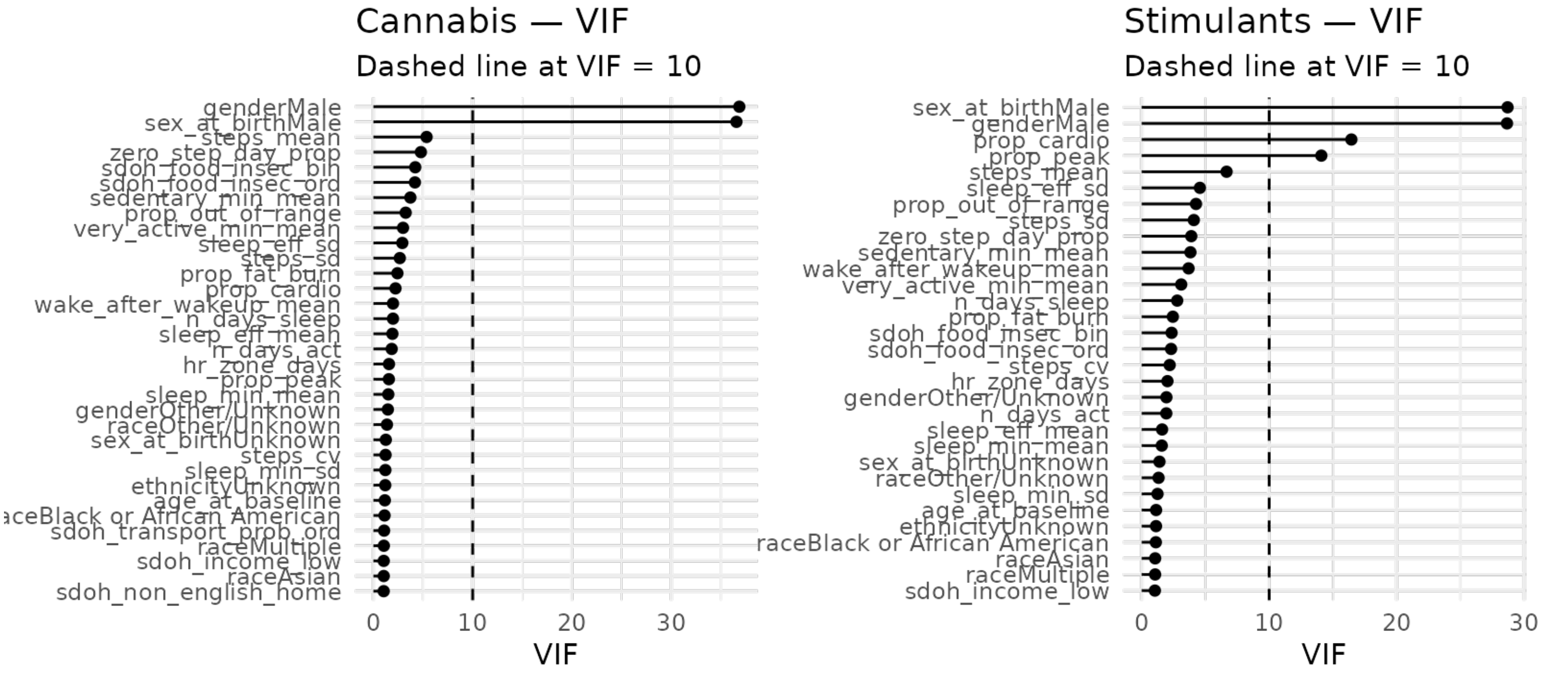

Lollipop plots display VIF values for each predictor across the Cannabis and Stimulant cohorts. The vertical dashed line marks the common threshold of VIF = 10, above which multicollinearity is typically considered problematic. Across cohorts, a small number of predictors exceed this threshold (Cannabis = 2, Stimulants = 4), primarily involving highly correlated wearable-derived variables. Despite these localized high-VIF features, the majority of predictors fall well below thresholds of concern, suggesting that collinearity was moderate and localized rather than widespread.

Supplementary Material

Supplementary Figure S3. Expanded Model Evaluation Across Cohorts

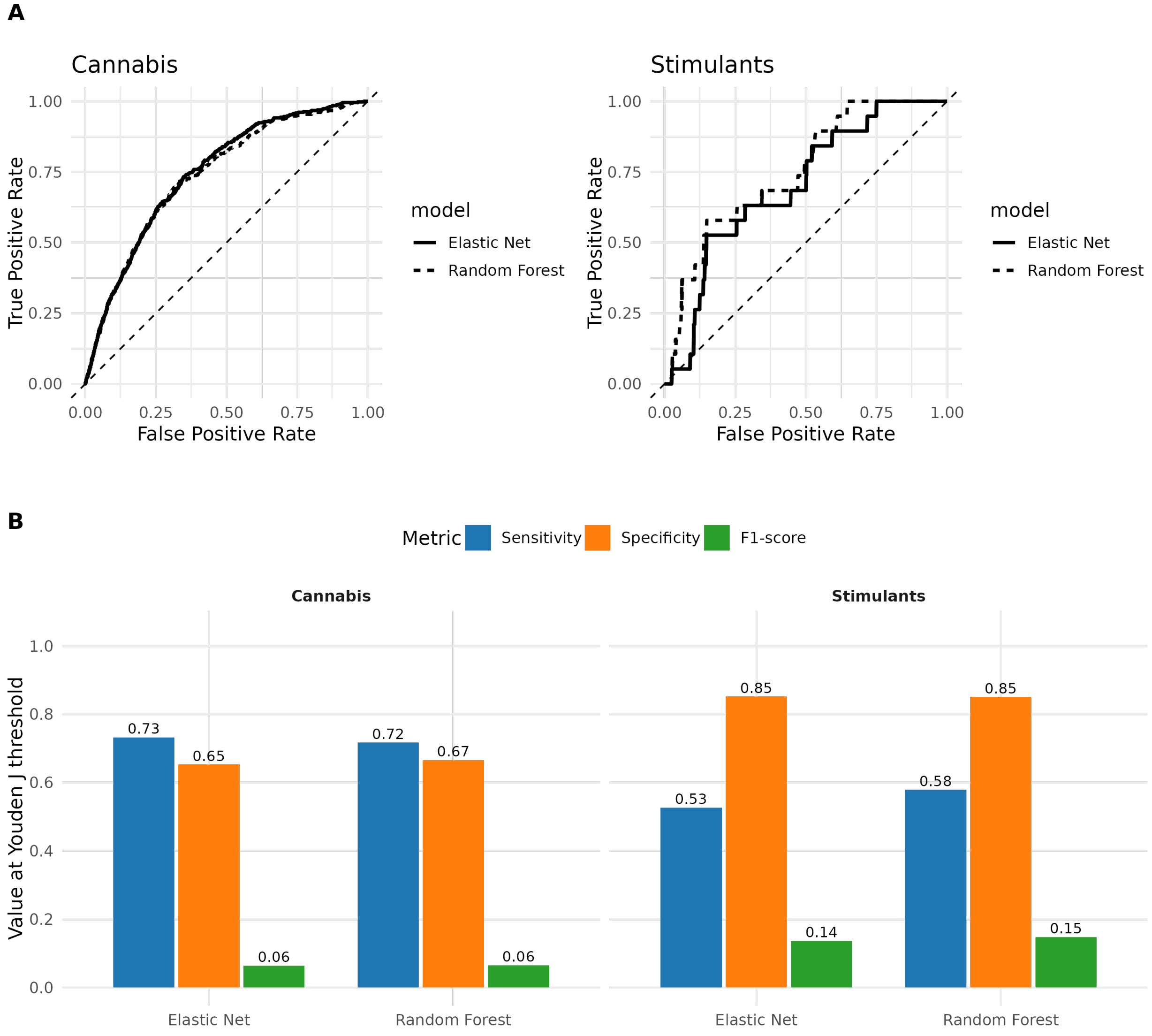

(A) ROC curves comparing elastic net and random forest for Cannabis and Stimulants; shaded bands show 95% CIs for AUC. Between-model AUC differences within each cohort were tested using the DeLong test.
(B) Threshold-dependent metrics on the independent test sets, evaluated at a fixed threshold of 0.50 and at the Youden J threshold.

Supplementary Material

Bars show Sensitivity (blue), Specificity (orange), and F1-score (green); numeric labels above the green bars report F1 values. Cohort abbreviations: Cannabis, Stimulants.

Supplementary Figure S4. Precision–Recall Curves for Stimulant-Use Disorder Prediction Across Flagged, Primary, and Strict Cohort Specifications

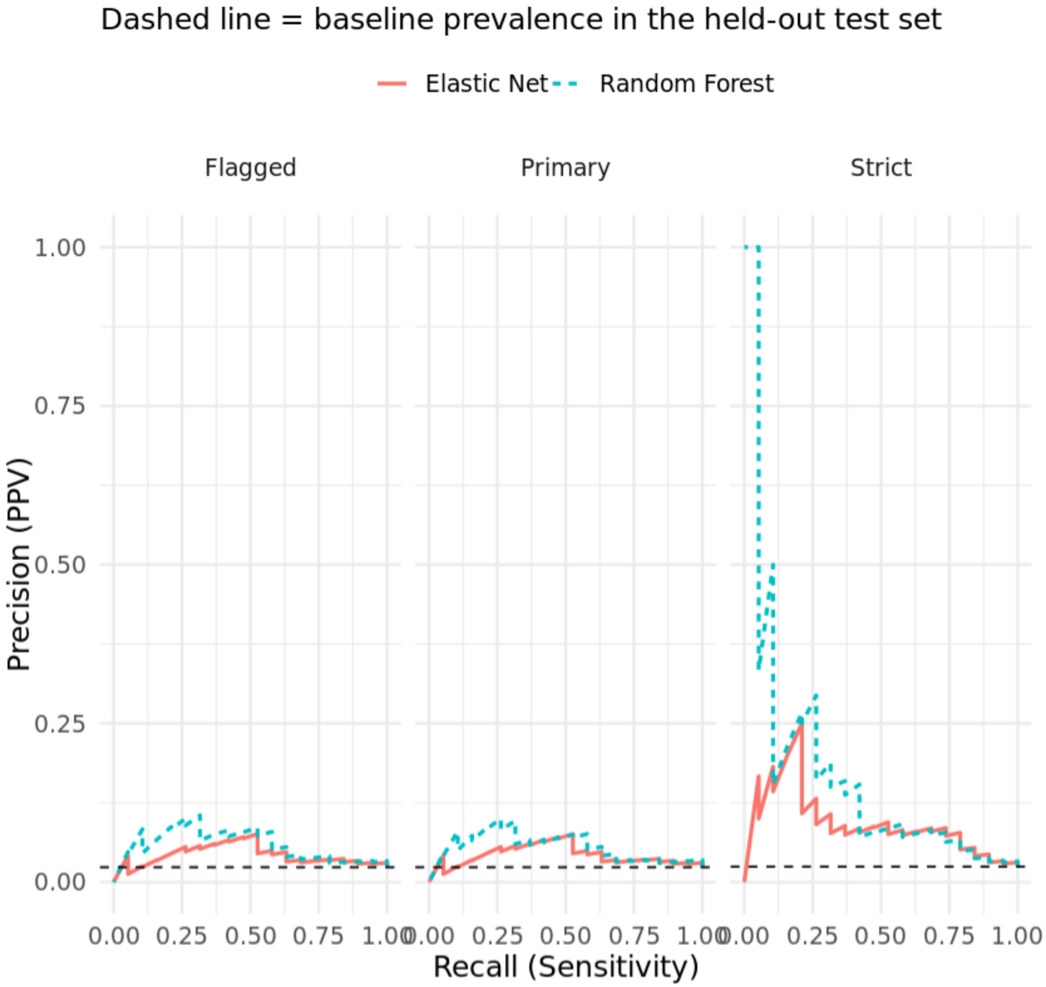

Precision–recall curves for stimulant-use disorder (Stimulant SUD) prediction across the Flagged, Primary, and Strict cohort specifications. Elastic net (red) and random forest (teal) models are shown. The dashed horizontal line indicates the baseline

prevalence in the held-out test set (~2.3%). Precision increases most notably in the Strict cohort specification, consistent with reduced exposure misclassification and greater case definition specificity.

Supplementary Figure S5. Elastic Net Coefficients for the Strict Stimulant Cohort

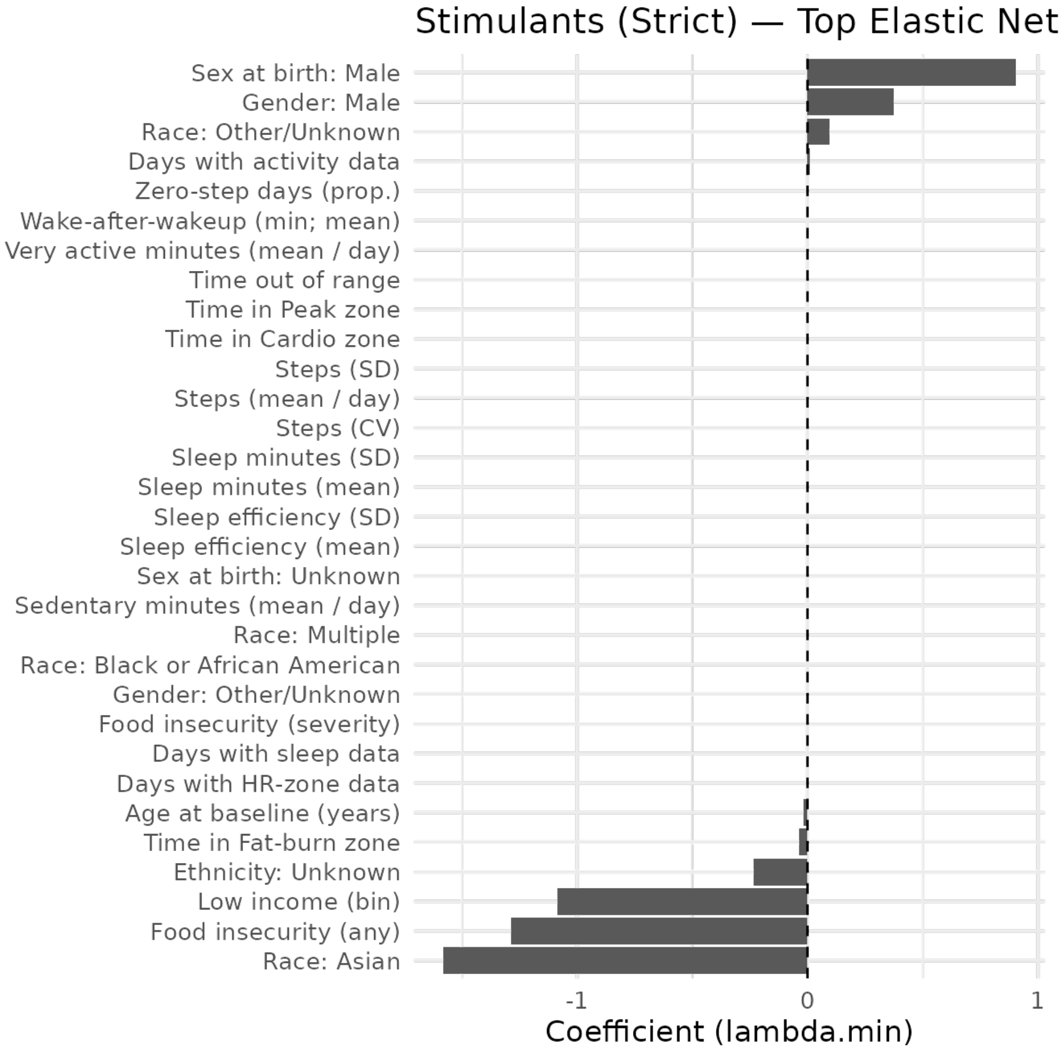

Supplementary Material

Standardized regression coefficients from the elastic net model in the Strict stimulant cohort (excluding participants with likely therapeutic stimulant exposure). Positive coefficients indicate higher predicted risk of stimulant use disorder (SUD), while negative coefficients indicate lower predicted risk. The model selected ~10 non-zero predictors, primarily demographic and socioeconomic (SDoH) characteristics (e.g., low income, food insecurity, and race/ethnicity indicators), rather than Fitbit-derived activity or sleep features. This supports the interpretation that improved performance in the Strict cohort reflects a cleaner phenotype definition, not increased model complexity or reliance on behavioral tracking data.

Supplementary Figure S6. Model Performance Across Main, Correlation-Pruned, and PCA Analyses

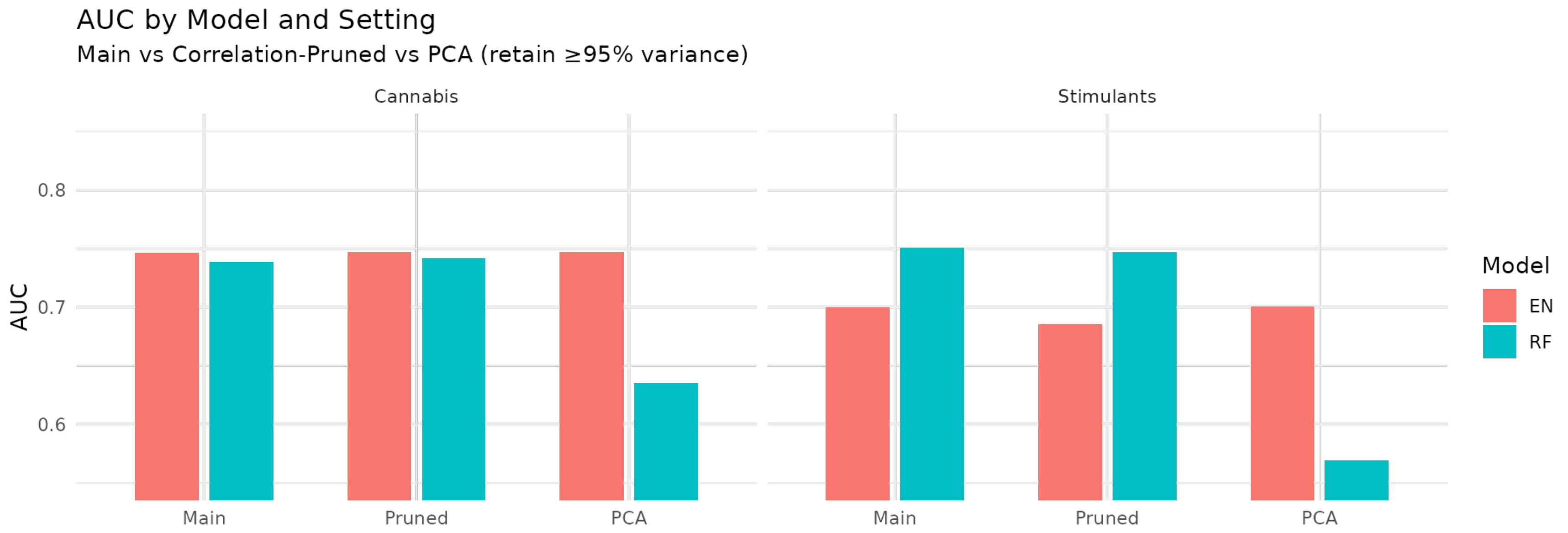

Bar plots compare model performance (AUC) for elastic net and random forest models across three modeling strategies: (1) Main Model: All predictors included; (2) Correlation-Pruned Model: One predictor removed from each pair with |r| ≥ 0.90; and (3) PCA Model: Principal component analysis applied, retaining components explaining ≥95% of variance. Results show that correlation-pruned models produce negligible changes in AUC for both elastic net and random forest models across both cohorts (Cannabis, Stimulants). PCA preserves performance for elastic net but substantially reduces AUC for random forest, suggesting that elastic net is robust to multicollinearity while tree-based models are more sensitive to feature redundancy.

Supplementary Material

Supplementary Figure S7. Model Error Rates (FPR, FNR) by Race

(a) Cannabis

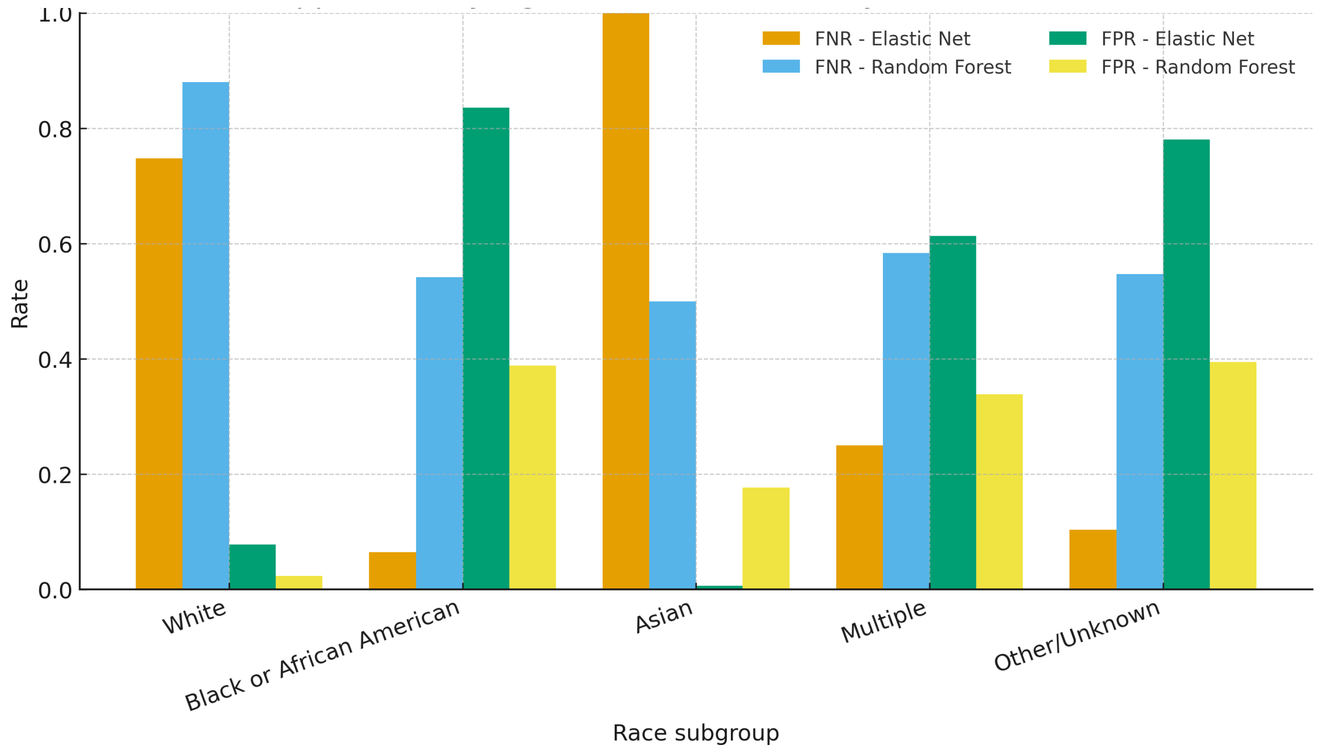

(b) Stimulants

Supplementary Material

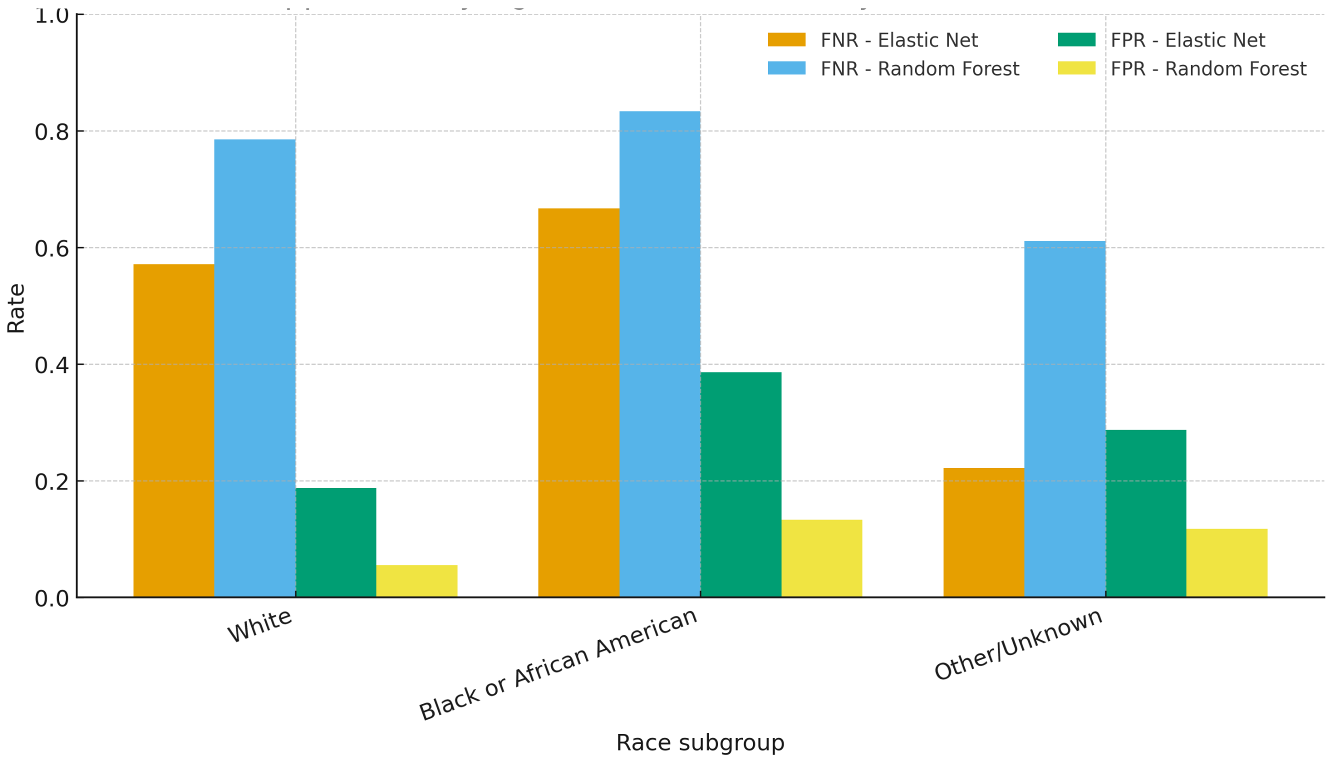

False negative rates (FNR) and false positive rates (FPR) are shown for each racial subgroup for the cannabis (top panel) and stimulant (bottom panel) cohorts, comparing elastic net and random forest models. FNR reflects the proportion of true cases not identified by the model (missed risk), while FPR reflects the proportion of non-cases incorrectly flagged (over-identification). For the cannabis cohort, elastic net exhibits notably higher FPR in Black, Multiple, and Other/Unknown groups, while random forest reduces FPR but at the cost of higher FNR in smaller subgroups (e.g., Asian). In the stimulant cohort, subgroup differences are present but less pronounced overall, though estimates are less stable due to low event counts and limited sample size. These patterns highlight trade-offs between sensitivity and specificity across racial groups and the importance of threshold and model choice when evaluating fairness in low-prevalence settings.

Supplementary Material

Supplementary Figure S8. Predicted Positive Rates (PPR) by Race
(a) Cannabis

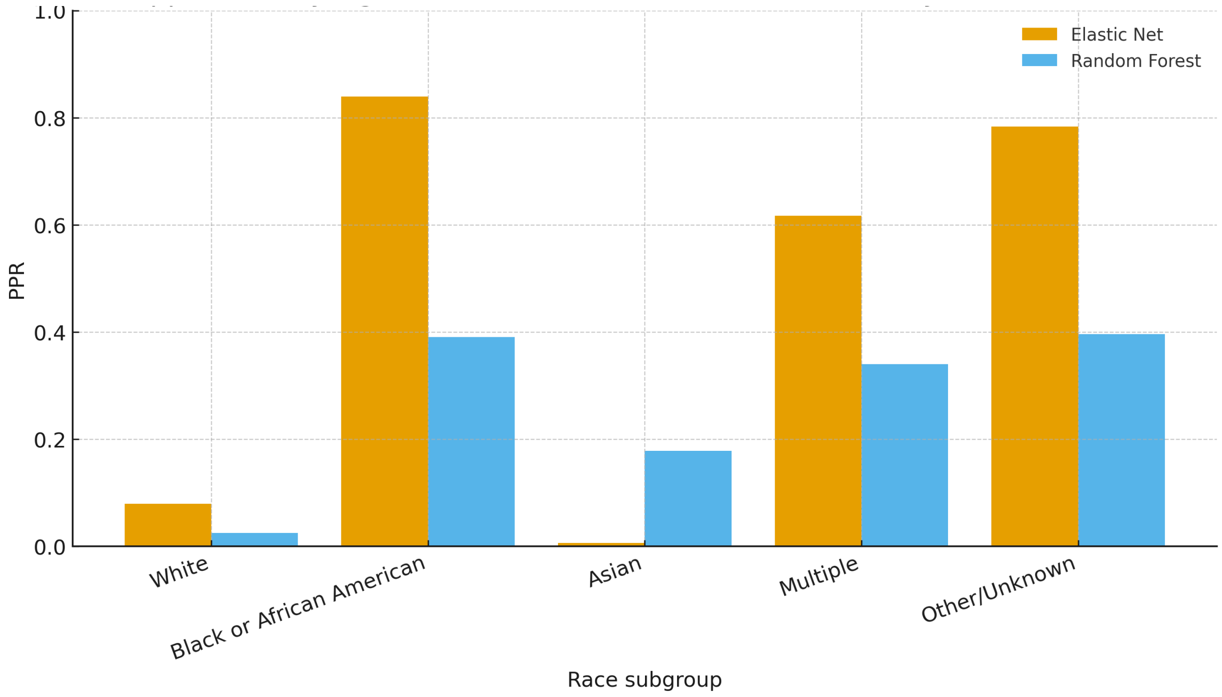

Supplementary Material

(b) Stimulants

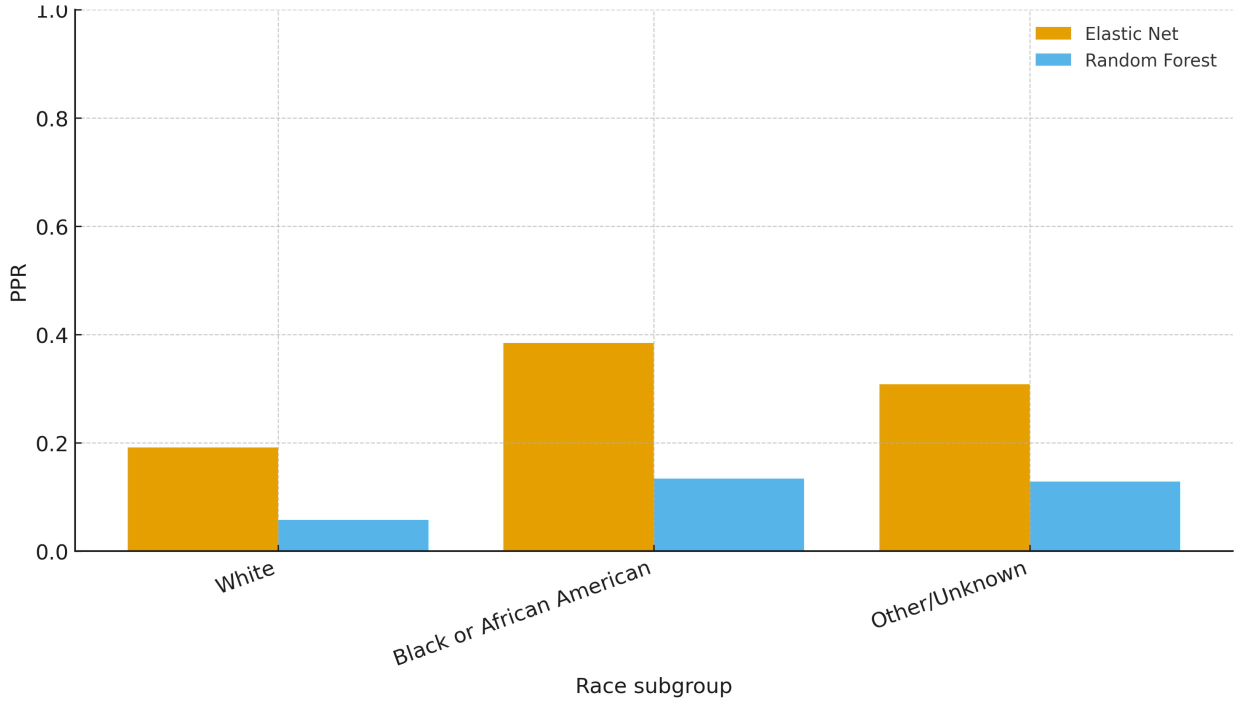

Predicted positive rates (PPR)—the proportion of individuals flagged as high-risk—are shown for each racial subgroup for the cannabis (top panel) and stimulant (bottom panel) cohorts, comparing elastic net and random forest models at the validation-selected operating threshold. Higher PPR indicates more individuals being identified for potential follow-up or intervention. For cannabis models, elastic net shows substantially higher PPR in Black, Multiple, and Other/Unknown subgroups compared to White and Asian subgroups, while random forest reduces overall PPR but retains the same subgroup pattern. For stimulant models, subgroup differences are present but smaller in magnitude, though estimates remain less stable due to low case counts. Together with the error rate results (**Supplementary Figure S7**), these patterns show that cannabis models—particularly elastic net—tend to flag some minoritized groups more frequently, underscoring the importance of evaluating model fairness in low-prevalence prediction settings.
